# RNA-Seq analysis of differential gene expression in *Betula luminifera* xylem during the early stages of tension wood formation

**DOI:** 10.7717/peerj.5427

**Published:** 2018-08-21

**Authors:** Miaomiao Cai, Huahong Huang, Fei Ni, Zaikang Tong, Erpei Lin, Muyuan Zhu

**Affiliations:** 1 The State Key Laboratory of Subtropical Silviculture, Institute of Biotechnology, College of Forestry and Biotechnology, Zhejiang A&F University, Hangzhou, China; 2 Key Laboratory for Cell and Gene Engineering of Zhejiang Province, Institute of Genetics, College of Life Sciences, Zhejiang University, Hangzhou, China

**Keywords:** RNA-Seq, Transcriptome, *Betula luminifera*, Tension wood formation, Auxin signaling pathway, Cellulose and lignin biosynthesis

## Abstract

**Background:**

* Betula luminifera* H. Winkler, which is widely distributed in southern China, is an economically important broadleaf tree species. However, little genomic information of *B*. *luminifera* is available, and little is known about the molecular mechanisms of wood formation in this species. Meanwhile, few efforts have focused on investigating the early transcriptional changes during tension wood formation in woody plants.

**Results:**

A reference transcriptome dataset was first generated containing 45,700 Unigenes, and 35,135 (76.9%) Unigenes were annotated by a BLAST similarity search against four public databases. Then, based on an anatomical investigation, the global gene expression changes during the early stages of tension wood formation were analyzed. Gene expression profiling showed that a total of 13,273 Unigenes were differentially regulated during the early stages of tension wood formation. Most genes involved in cellulose and lignin biosynthesis were highlighted to reveal their biological importance in tension wood formation. In addition, the transcription levels of many genes involved in the auxin response pathway were significantly changed during the early stages of tension wood formation. Furthermore, 18 TFs co-expressed with key enzymes of cellulose synthesis were identified.

**Conclusions:**

Our results revealed the transcriptional changes associated with TW formation and identified potential key genes in the regulation of this process. These results will help to dissect the molecular mechanism of wood formation and provide key candidate genes for marker-assisted selection in *B. luminifera*.

## Introduction

Wood formation is a complex biological process that involves a series of sequential steps, including cell division from the vascular cambium, cell function specification, secondary cell wall deposition, programmed cell death and heartwood formation. The process is regulated not only by endogenous factors such as sugars and plant hormones (Björklund et al., 2007; Brackmann et al., 2018; Sorce et al., 2013), but also by environmental stimuli (Begum et al., 2013; Moyle et al., 2002). For example, reaction wood, a special wood made of specialized xylem cells, is induced by gravistimulation and is associated with complicated transcriptional and hormonal regulation (Felten et al., 2018; Wang et al., 2017; Groover, 2016; Gerttula et al., 2015). The formation and properties of reaction wood in gymnosperm differ from those in angiosperm. Therefore, there are two types of reaction wood: compression wood (CW) in gymnosperms and tension wood (TW) in angiosperms. CW develops on the lower side of leaning stems and branches, whereas TW occurs on the upper side of leaning stems and branches. In comparison to normal wood (NW), CW has a higher proportion of lignin, whereas TW has a higher proportion of cellulose (Du, Uno & Yamamoto, 2004; Funada et al., 2008; Jin & Kwon, 2009). The formation of reaction wood has widely been used as an experimental system to dissect the molecular mechanisms of wood formation. Gene expression profiles during reaction wood formation have been studied using microarrays developed from EST databases, and several key candidate genes involved in reaction wood formation have been identified (Andersson-Gunneras et al., 2006; Paux et al., 2005; Villalobos et al., 2012).

Previous studies on gene expression profiles in TW formation were generally conducted only at one time point, which was usually several weeks or months after applying a stimulus, and these studies have failed to comprehensively analyze the effects of differentially expressed genes involved in reaction wood formation (Andersson-Gunneras et al., 2006; Oakley et al., 2007; Yamashita, Yoshida & Yamamoto, 2009). However, the study in *Eucalyptus globulus* has revealed that the genes activated at the early stages of TW formation could have a significant impact on the properties of the wood formed later on (Paux et al., 2005). It is therefore important to study gene expression profiles at a number of time points in the early stages of reaction wood formation.

The breakthroughs in second-generation sequencing technologies, especially for Illumina RNA-Seq, have offered new opportunities for comprehensive transcriptomic analyses in nonmodel tree species. Owing to the high throughput and ability to detect rare transcripts, RNA-Seq and digital gene expression profiling (DGE) have been applied to explore metabolic mechanisms related to the growth and product quality of some nonmodel plants, and the results demonstrate their potential in the discovery of key candidate genes controlling economic traits (Chen, Chen & Zhang, 2015; Feng et al., 2012; Hao et al., 2011; Mutasa-Gottgens et al., 2012; Tao et al., 2012; Wang et al., 2014).

*Betula luminifera* H. Winkler, a broadleaf tree species, is widely distributed in 14 provinces of southern China. Because of its desirable wood properties and fast growth rate, this tree species has been widely grown to produce timber for manufacturing high-quality furniture, wood veneers and solid wood flooring. In addition to its high economic value, *B. luminifera* has a relatively short juvenile period, and many germplasms of *B. luminifera* start flowering in 18 months. Such a short life cycle could speed up the breeding progress, making *B. luminifera* an ideal tree species for the genetic improvement of wood properties of native forest trees. However, little genome or transcriptome data are available, which has hindered progress towards the understanding of the molecular mechanisms underlying wood formation and the improvement of wood quality. To provide comprehensive genome-wide transcript profiling in TW formation and to identify the key genes involved in the wood formation of *B*. *luminifera*, a reference transcriptome from multiple organs and specific tissues was obtained by paired-end sequencing, and then the reference transcriptome was used to analyze the transcripts derived from the developing xylem of TW from short-time bending treatments. The high-resolution transcriptome profiles provided insights into the genes involved in the early stages of TW formation, particularly for those responsible for the biosynthesis of cellulose and lignin, and for auxin signaling.

## Materials and Methods

### Plant materials

*B. luminifera* clone (1V25-2) plants used in this study were grown in an environmentally controlled greenhouse of Zhejiang A&F University, Hangzhou, China. The greenhouse was set to a 16-h light/8-h dark cycle, and the air temperature was held between 20 and 28 °C. For reference transcriptome sequencing, different samples were collected from one-year-old trees at 3 pm between April and May 2014. Among these samples, male and female inflorescences and mature leaves were sampled from the upper crown on 4th April and 15th April 2014, respectively. Roots from hydroponic plants and the lignified stems from the upright current-year branches of the upper crown were collected on 12th May 2014. For the RNA-Seq analysis of TW formation, TW induction was performed through the bending method as described by Huang et al. (2014) and Paux et al. (2005). Four time-points, including 0 h, 6 h, 48 h and 7 d, were set up for bending treatment. At each time point, five straight one-year-old plants were bended at the 15th internode (counted from the top) to a 45° angle and fixed with ropes to induce TW formation. The 7 d bending treatment was first started at 3 pm on 12th May 2014, the 48 h bending treatment was then started at 3 pm on 17th May, and the 6 h bending treatment was started 9 am on 19th May. All of the bending treatments ended at 3 pm on 19th May, and the xylem samples were collected immediately after the completion of the bending treatment. The xylem samples from five straight plants without bending treatment (0 h) were also collected at the same time and taken as control (NW). The xylem tissues (approximately 2 mm in thickness) were scraped from the upper side of the bent stems, as well as from the equivalent position of the control after the removal of the bark. To identify the genes preferentially expressed in developing xylem, leaf blades (Leaf) were also prepared from mature leaves by removal of the central vein. All samples used for RNA isolation were immediately frozen in liquid nitrogen and stored at −80 °C.

### Anatomical analysis

Xylem samples were collected after the bending treatment for six months. To determine the G-layers for the presence of TW, 20 µm thick sections were examined using scanning electron microscopy (Foston et al., 2011), and 10 µm sections in thickness were double-stained with Safranin O and Fast Green FCF. The lignified walls are stained red with Safranin O, whereas Fast Green stains the pure cellulosic G-layer blue–green. Furthermore, fluorescence microscopy was used to directly observe the autofluorescence of lignin in unstained sections (Selig et al., 2012). The wall thickness of fiber was measured using a modification of Franklin’s method (McLaughlin & Schuck, 1991). At least five different plants were used, and 50 fibers from each plant were measured using a light microscope, and the data were analyzed for significant differences using SPSS 19.0 (IBM) with Student’s *t* test.

### Measurements of sucrose, fructose and IAA

The Sucrose/Fructose/D-Glucose assay kit (Megazyme, Wicklow, Ireland) was used to determine sucrose and fructose contents by following the manufacturer’s instructions. The contents of indole-3-acetic acid (IAA) were determined as described by Yang et al. (2001). Each treatment had five biological replicates. The data were analyzed for significant differences using SPSS 19.0 (IBM) with Student’s t test.

### Total RNA isolation

Total RNA from the different tissues was extracted with the PureLink™ Plant RNA Reagent (Invitrogen, CA, USA) according to the manufacturer’s protocol. To remove DNA contaminants, total RNA samples were then treated with RQ1 RNase-free DNase (Promega, WI, USA). RNA integrity was determined using a 2100 Bioanalyzer RNA Nanochip (Agilent, CA, USA) with an RNA Integrity Number (RIN) value of more than 7.0. The RNA concentration was determined using a NanoDrop ND-1000 spectrophotometer (Nano-Drop, DE, USA).

### Construction of a cDNA library and reference transcriptome sequencing

A mixture of equal amounts of RNA from mature leaves, stems, roots, inflorescences and TWs was used for reference transcriptome sequencing. The cDNA library with fragments between 200 and 700 bp was constructed using the Illumina kit according to the manufacturer’s instructions (Illumina, CA, USA). Transcriptome sequencing was performed using Illumina HiSeq 2000 (100-bp paired-end reads) at Beijing Genomics Institute (BGI, Shenzhen, China).

### Analysis of reference transcriptome sequence data

Raw reads were filtered to obtain high-quality clean reads by removing adaptor sequences, reads containing more than 5% ambiguous bases and low-quality reads containing more than 20% bases with a *Q*-value <10. The de novo assembly of clean reads was carried out with the short-read assembly program Trinity with default parameters (Grabherr et al., 2011). Trinity first combines reads with a certain length of overlap to form longer fragments, which are called contigs. Then, the reads are mapped back to contigs; with paired-end reads it is able to detect contigs from the same transcript and the distances between these contigs. Finally, Trinity connects the contigs and obtains sequences that cannot be extended on either end. Such sequences were defined as Unigenes and were used for a blastx search against four public protein databases with an *E*-value ≤1e−5. These protein databases included the NCBI non-redundant proteins (NR) database (http://www.ncbi.nlm.nih.gov), the Swiss-Prot protein database (http://www.expasy.ch/sprot), the Kyoto Encyclopedia of Genes and Genomes (KEGG) database (http://www.genome.jp/kegg), and the Clusters of Orthologous Groups of proteins (COG) database (http://www.ncbi.nlm.nih.gov/COG). Unigenes with highest sequence similarity were retrieved for subsequent analysis. Based on NR annotation, Gene Ontology (GO) functional classification was obtained by WEGO (http://wego.genomics.org.cn/) (Kotake et al., 2011) via GO terms assigned by Blast2GO (version 2.3.5) with an *E*-value threshold of 1e−5 (Karpinska et al., 2004). The possible functions of Unigenes were also predicted and classified by aligning to the COG database with an *E*-value threshold of 1e−5. Pathway assignments were carried out according to the KEGG database using blastx with an *E*-value threshold of 1e−5.

### Profiling differentially expressed genes

The RNA was isolated from the xylem sample of each plant, and then the RNA samples from the five individual plants of each time point were equally mixed to construct four TW cDNA libraries. Another cDNA library using leaf RNA samples derived from leaf blades without the central vein was also constructed to identify the xylem-specific genes. A total of five cDNA libraries were prepared according to the manufacturer’s instructions (Illumina, San Diego, CA, USA). These cDNA libraries were sequenced via Illumina HiSeq2000 (1 ×50-bp read length) by BGI in Shenzhen, China. Raw reads were first filtered by removing adaptors, reads containing more than 10% ambiguous bases, and low-quality reads containing more than 50% bases with a *Q*-value ≤5. All clean reads were mapped to the reference transcriptome sequences described above using SOAPaligner 2.0 with the parameters “-m 0 –x 1,000 –s 40 –l 35 –v 3 –r 2” (Li et al., 2009). Then, the number of mapped clean reads for each Unigene was calculated and normalized to a Reads Per Kilobase per Million clean reads (RPKM) value (Mortazavi et al., 2008). Based on the RPKM values differentially expressed Unigenes during the early stages of TW formation were identified according to the report of Audic and Claverie (Audic & Claverie, 1997). The false discovery rate (FDR) was calculated via SAS 8.0 and was used to identify the *p*-value threshold in multiple tests and analyses (Benjamini & Yekutieli, 2001). Compared to NW, the differentially expressed genes (DEGs) during the early stages of TW formation were screened with the threshold of FDR <0.001 and the absolute value of log_2_(ratio) ≥1. Overall expression patterns of Unigenes, excluding those with nonsignificant expression changes or with changes over 32-fold, were visualized in 3D with MATLAB (R2012). Among the DEGs, those that showed up- or down-regulated expression at all tension time-points were taken as up- or down-regulated Unigenes, respectively. Then, the GO functional classifications of the up-regulated and down-regulated Unigenes were performed using WEGO.

In addition, *B. luminifera* Unigenes, which are potentially involved in the cellulose and lignin biosynthetic pathways and auxin regulation, were identified according to the method described by Shi et al. (2011). To reflect dynamic changes and absolute expression patterns at the early stages of TW formation, eight different colors were used to represent different RPKM values of these Unigenes. The RPKM values were converted to logarithm base 2, and clustering was performed using MultiExperiment Viewer (MeV) (version 4.9.0) with the algorithm “K Means Clustering”. Cellulose and lignin biosynthetic pathways were produced manually as described by Huang et al. (2012).

To verify the quality of assembled Unigenes, 20 genes involved in cellulose and lignin biosynthesis (Table S1) were isolated using the SMARTer™ RACE cDNA amplification kit (Clontech, CA, USA) according to the manufacturer’s instructions. Based on the sequences of RACE cloning, full-length cDNAs of these genes were cloned by RT-PCRs using the PrimeScript^^®^^RT-PCR kit (TaKaRa, Dalian, China). The primers used are listed in Table S2. The open read frames (ORFs) of the putative full-length cDNA sequences were predicted using the online ORF finder program (https://www.ncbi.nlm.nih.gov/orffinder/), and the amino acids were deduced using DNASTAR 7.0. Phylogenetic analysis of genes was performed using MEGA4.0 (Tamura et al., 2007). The GenBank accession numbers of other gene sequences are listed in Table S3 . To evaluate the quality of assembled Unigenes, full-length sequences of these cloned genes were aligned with related Unigenes using the program AlignX (Invitrogen, CA, USA).

### Co-expression analysis

Co-expressed gene networks were constructed by means of Mutual Rank (MR). First, Unigenes supported by less than 10 reads in each subsample were filtered, and the sample redundancy was calculated with RPKM. Then, PCCs (Pearson’s correlation coefficients) between gene expression patterns were calculated following known formulas (http://atted.jp/help/coex_cal.shtml) and were converted into MRs with the method described by (Obayashi et al., 2007). For each guide gene, the top 20 related genes were selected to produce the co-expressed gene relationships. Finally, co-expression networks were visualized using Cytoscape 2.8.3 (Smoot et al., 2011).

### Quantitative RT-PCR validation and expression analysis

Gene expression patterns in different tissues were analyzed by qRT-PCR using the CFX96™ Real-Time PCR Detection System (Bio-Rad). Twenty genes involved in cellulose and lignin biosynthesis and the IAA signaling pathway were validated. All PCR reactions were performed with five replicates. The actin gene (GenBank accession number: FJ410442), which is stably expressed in different tissues/organs, was chosen as an internal reference for normalization. The primers used for qRT-PCR are listed in Table S2.

## Results

### Reference transcriptome analysis of paired-end sequencing data

First, a reference transcriptome of *B. luminifera* was generated by paired-end sequencing. A total of 70,358,340 clean reads with an average length of 90 bp were obtained and assembled into 45,700 Unigenes with an average length of 775 bp and a combined length of 35.4 Mb. Of all the Unigenes, 23,149 (50.7%) were over 500 bp long, and 6,025 were longer than 1,500 bp (Fig. S1A). The mean sequencing depth of the Unigenes was 34.02, while 87.9% of the Unigenes were mapped by more than 10 reads, and 39.1% of them were mapped by more than 100 reads (Fig. S1B). To verify the quality of the assembled Unigenes, 20 genes involved in cellulose and lignin biosynthesis were cloned and then compared with related Unigenes by sequence alignment as shown in Table S1, 27 Unigenes were found to cover whole or partial regions of the cloned genes with sequence identities over 98%. For example, the Unigene 4901.6 covered 99.3% of the *BlCesA2* with a sequence identity up to 99.6%. This indicated that the assembled Unigenes of the *B. luminifera* transcriptome were high in quality. Therefore, they could be used as a reference transcriptome for transcriptomic profiling with RNA-Seq.

### Functional annotation and classification of the reference transcriptome

**Figure 1:**
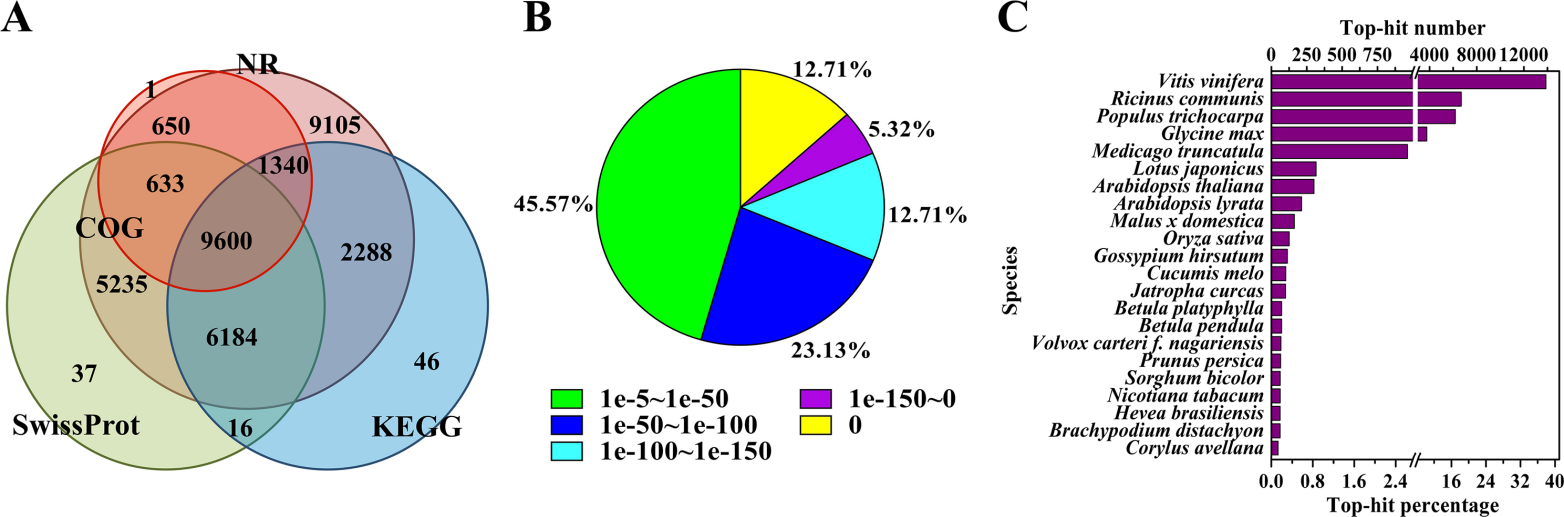
Characteristics of the homology search of *B. luminifera* Unigenes. (A) Venn diagram of the number of Unigenes annotated by BLASTX against protein databases (the *E*-value threshold was set at 1e−5). The numbers in the circles indicate the number of Unigenes annotated by single or multiple databases. (B) *E*-value distribution of the top BLASTX hits against the NR database for each Unigene. (C) Number and percentage of Unigenes matching the top 22 species using BLASTX against the NR database.

To annotate the assembled Unigenes, sequence similarity searches were performed against four public databases. A total of 35,135 (76.9%) sequences showed significant similarity to known proteins in at least one of the searched databases, and 9,600 (21.0%) had significant matches in all four databases (Fig. 1A). According to the NR annotations, 81.6% of the mapped sequences showed strong homology (*E*-value <1e−20), and 54.4% showed very strong homology (*E*-value <1e−50) to available plant protein sequences (Fig. 1B). As shown in Fig. 1C, over 87% of the Unigenes could be annotated with proteins from four top-hit species, i.e., *Vitis vinifera*, *Ricinus communis*, *Populus trichocarpa*, and *Glycine max*. Based on the GO annotations, 27,531 Unigenes were assigned to 59 subcategories (Fig. S2). In the three main GO categories (cellular component, biological process and molecular function), ‘cell’, ‘metabolic process’ and ‘catalytic activity’ were the most abundant groups, respectively. A total of 18,125 Unigenes belonging to the ‘metabolic process’ group could be used to identify the genes involved in the secondary growth of the cell wall during wood formation.

Moreover, the assembled Unigenes were compared against the COG database for in-depth analysis of phylogenetically widespread domain families. Of all the 45,700 Unigenes, 12,224 had a COG classification. Among the 25 COG categories, ‘general function prediction only’ (4,075 Unigenes, 33.3%) and ‘nuclear structure’ (three Unigenes, 0.02%) represented the largest and smallest groups respectively (Fig. S3). It is noteworthy that 1,125 Unigenes were assigned to the category of cell wall/membrane/envelope biogenesis.

Pathway-based analysis, performed by searching against the KEGG database, helps to further understand the biological functions and interactions of genes. There were 19,474 Unigenes mapped to 128 KEGG pathways. The pathways with the highest Unigene representation were metabolic pathways (Ko01100, 4,653 Unigenes, 23.89%), followed by secondary metabolites (Ko01110, 2,199 Unigenes, 11.29%) and plant-pathogen interactions (Ko04626, 1,176 Unigenes, 6.04%) (Table S4). These annotations would be a valuable resource for further research on specific processes, functions and pathways of wood formation. As an example, phenylpropanoid biosynthesis, an important pathway associated with lignification, was examined. Overall, 384 Unigenes were mapped to this pathway, which contains more than 50% of the known enzymes (Fig. S4).

**Figure 2:**
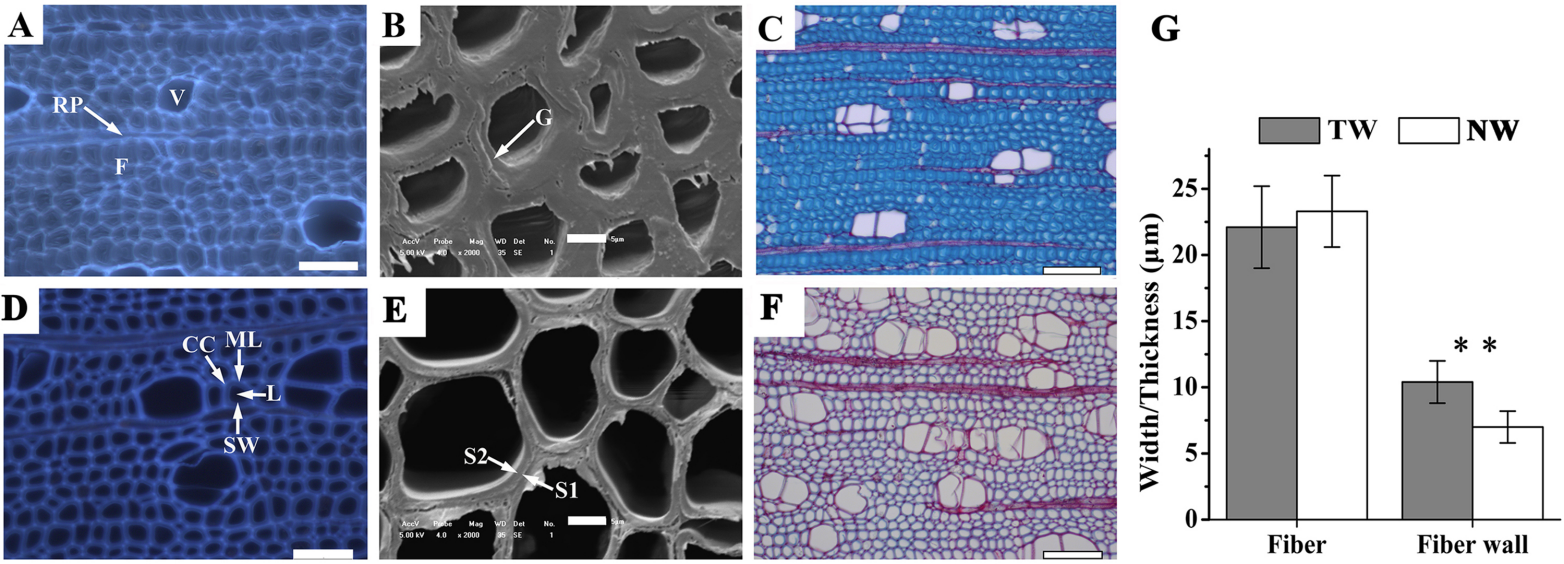
Tension wood (TW) formation in *B. luminifera* (A) and (D) Autofluorescence image of the xylem cross-section of TW and NW, respectively. V, vessel; F, fiber; RP, ray parenchyma; ML, middle lamella; CC, cell corner; L, lumen; SW, secondary cell wall. Scale bars = 50.0 µm. (B) and (E) Scanning electron microscopy images of the xylem cross-sections of TW and NW, respectively. G, gelatinous layer; S1, S2, layers of the secondary cell wall. Scale bars = 5.0 µm. (C) and (F) Sections of TW and NW double-stained with Safranin O and Fast Green FCF, respectively. Scale bars = 100.0 µm. (G) Width and wall thickness of wood fiber. ** indicates a significant difference at 1%.

### Anatomical characteristics of bending-induced tension wood

Xylem samples of *B. luminifera* were taken after six months of bending and were transversely sectioned. The lumen diameters of fiber cells in TW were obviously smaller than those in NW, while the fiber cell walls in TW were much thicker (Figs. 2A, 2B, 2D and 2E). Further measurement indicated that no significant difference was detected in the fiber width between TW and NW, but the mean wall thickness of TW was 1.5-fold that of NW (Fig. 2G). This means that cell wall thickening mainly occurred on one side of the fiber lumen. Transverse sections were also double-stained with Safranin O and Fast Green FCF, by which the lignin of the cell wall is stained red with Safranin O, and celluloses are stained blue–green with Fast Green. As shown in Fig. 2C, the secondary cell walls (SCWs) in TW were almost all stained bluish green, whereas those in NW were almost all red (Fig. 2F). This indicated the presence of a cellulosic G-layer in TW. These findings were consistent with previous reports regarding other species, such as *Populus euramericana*, *Eucalyptus globulus*, *Eucalyptus gunnii* and *Fraxinus mandshurica*, where the G-fibers were formed under similar treatments (Jiang et al., 2009; Jourez, Riboux & Leclercq, 2001; Paux et al., 2005; Toghraie et al., 2006). In general, typical TW can be clearly generated when *B. luminifera* is under bending treatment.

### Changes in sucrose, fructose and IAA contents during the early stage of TW formation

**Figure 3:**
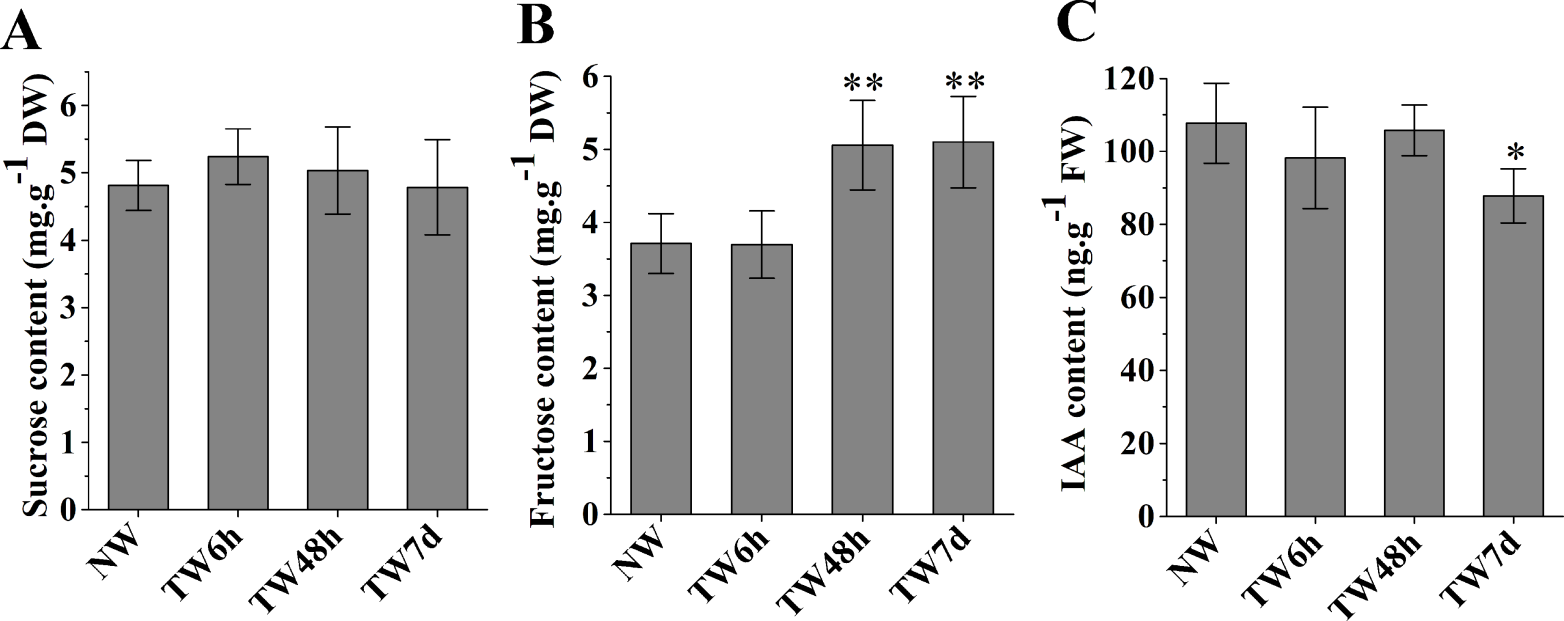
The contents of sucrose, fructose and IAA in developing xylem at different times of bending. (A) Sucrose content. (B) Fructose content. (C) IAA content. * and ** indicate significant differences at 5% and 1% compared with the control, respectively.

The hydrolysis of sucrose catalyzed by sucrose synthase (SUS) generates UDP-glucose to provide the substrate for cellulose biosynthesis. As shown in Fig. 3A, the sucrose contents at three time-points presented nonsignificant differences compared with NW. Fructose, another product of sucrose decomposition, was obviously accumulated in TW after 48 h bending, and the corresponding differences between TW and NW reached 1.34 and 1.39 mg g^−1^DW, respectively (Fig. 3B). Early studies also demonstrated that indole-3-acetic acid (IAA) was tightly involved in TW formation of hardwood species (Kennedy & Farrar, 1965; Leach & Wareing, 1967; Robnett & Morey, 1973). In comparison with NW, a significant reduction in the IAA concentration in TW was observed after 7 d of bending, but no obvious changes occurred after 6 h or 48 h of bending (Fig. 3C).

### Global transcriptome analysis during TW formation

#### Identification and annotation of differentially expressed genes (DEGs)

**Figure 4:**
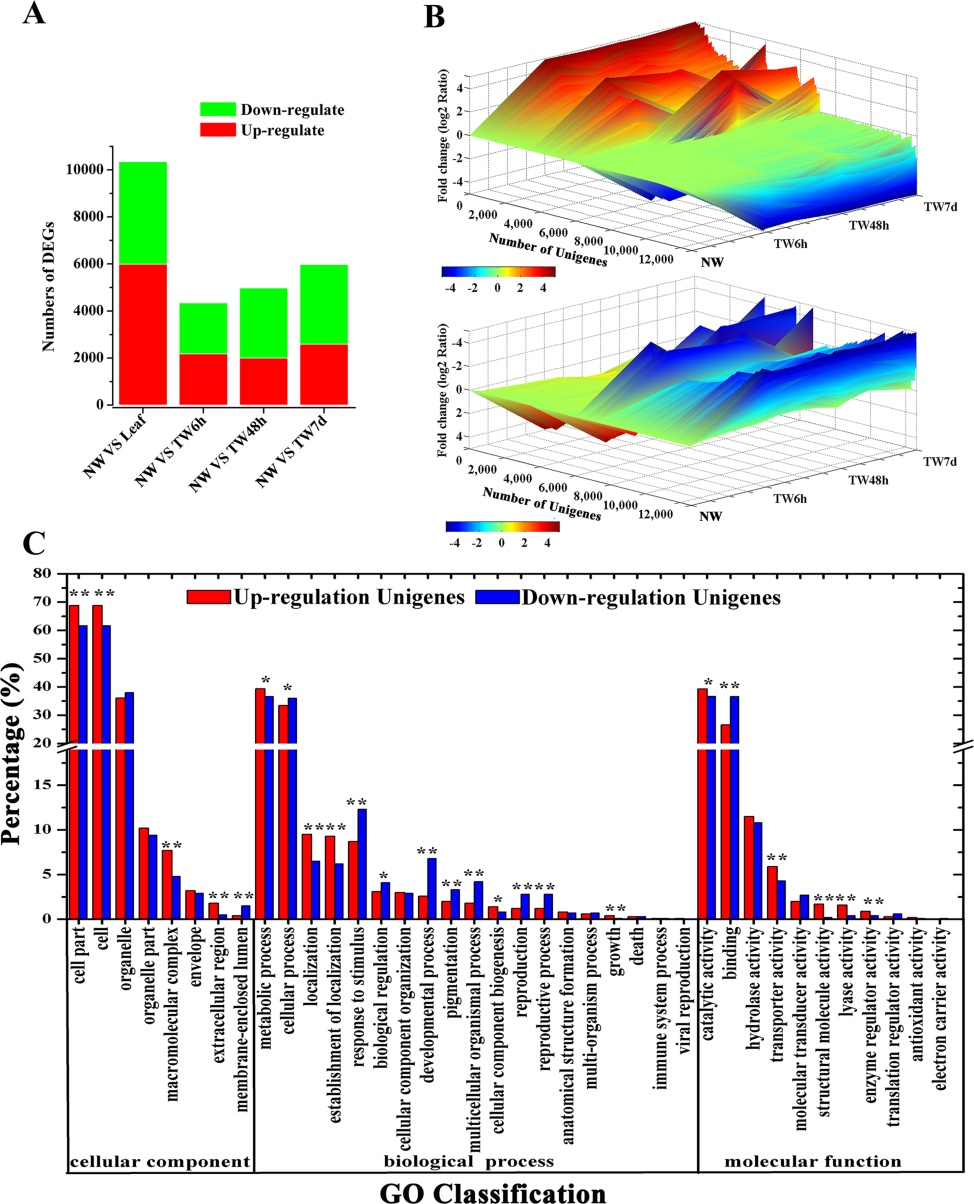
Unigene expression profiles during TW formation of *B. luminifera*. (A) Statistics of differentially expressed genes between different tissues. Up-(red) and downregulated (green) transcripts are quantified. (B) Overall expression profiles for the Unigenes differentially expressed in four different xylem libraries. (C) GO classification of up-regulated and down-regulated Unigenes during TW formation of *B. luminifera*. * and ** indicate significant differences at 5% and 1%, respectively.

Having generated a reference transcriptome for *B. luminifera*, our next goal was to perform quantitative measurements of the transcriptome in the developing xylem at the early stages of TW formation. To achieve this, the global gene expression was determined by RNA-Seq in different xylem samples, including 0 h (NW, normal wood) and three different tension woods (TW6 h, bent for 6 h; TW48 h, bent for 48 h; and TW7d, bent for 7 d). According to the RPKM and FDR methods (Audic & Claverie, 1997; Mortazavi et al., 2008), 21,621 Unigenes were expressed during TW formation, with 4,348, 4,983 and 5,977 Unigenes showing differential expression between NW and TW6 h, NW and TW48 h, and NW and TW7d, respectively (Fig. 4A). Taken together, 13,273 DEGs were found at the early stages of TW formation. Among these DEGs, 35.5% were also preferentially expressed in NW compared with the Leaf sample (Fig. 4A). The expression of DEGs exhibited multiple patterns, which are shown in Fig. 4B. During this process, 3,650 and 5,749 DEGs showed significantly up-regulated and down-regulated expression, respectively. It is worth noting that the lowly expressed Unigenes could have changes over 1,000-fold. The up-regulated and down-regulated Unigenes could be categorized into 38 GO functional groups (Fig. 4C). The GO terms of metabolic process and catalytic activity were two major responsive groups in which the numbers of up-regulated Unigenes were significantly higher than those of the down-regulated Unigenes, suggesting that metabolic processes were enhanced by the bending treatment.

**Figure 5:**
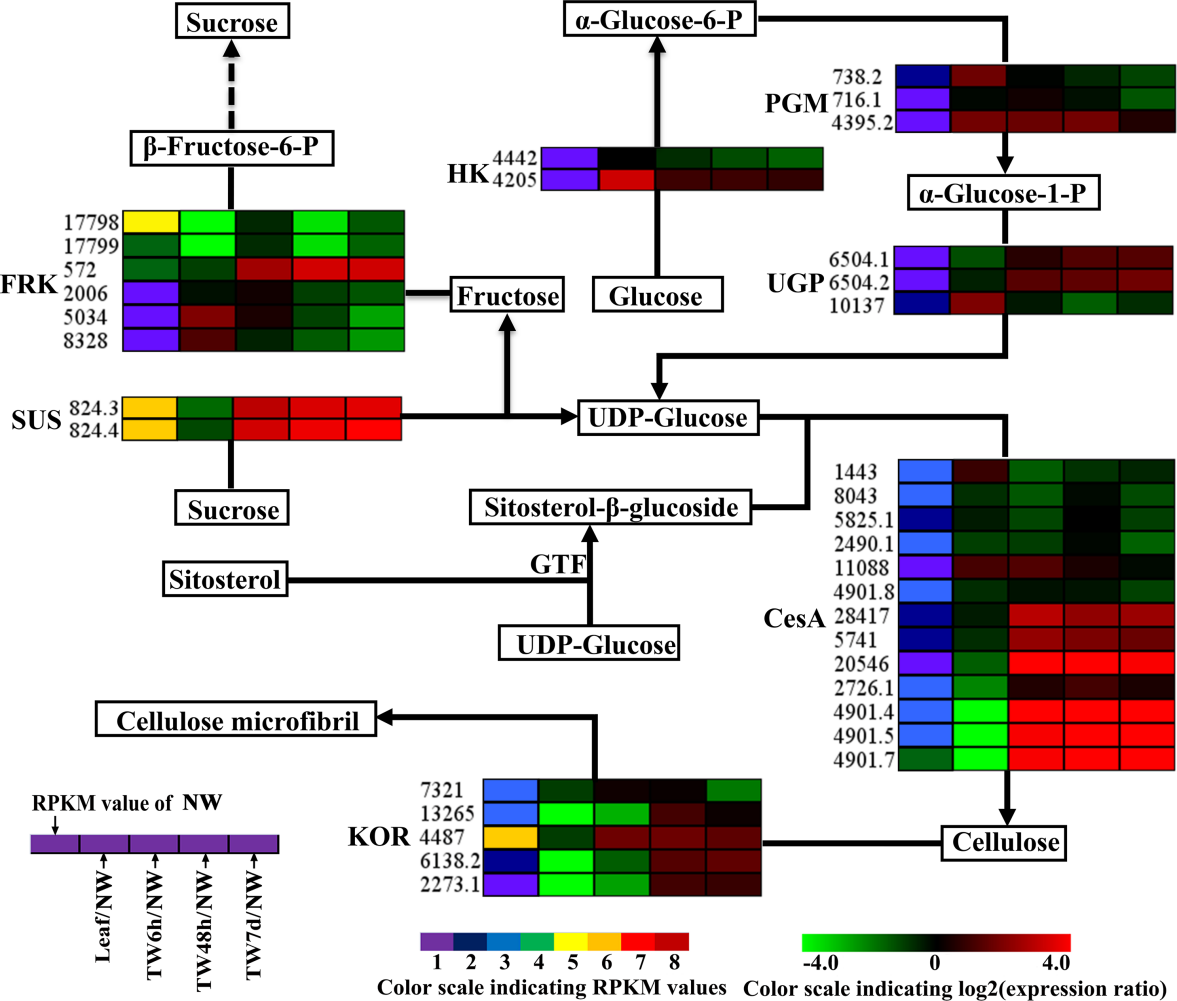
Expression patterns of DEGs involved in the cellulose biosynthesis pathway. Enzyme names, Unigene ids and expression patterns are indicated on each step. The expression pattern of each Unigene is shown by five grids, with the left representing the RPKM value of the NW sample and the second to fifth representing the relative log2 (expression ratio) for the leaf, TW6h, TW48h and TW7d samples, from left to right. The grids with eight different colors from purple to red show the absolute expression magnitude of the NW sample, with RPKM values of 0–10, 10–20, 20–40, 40–80, 80–160, 160–320, 320–640 and over 640.

**Figure 6:**
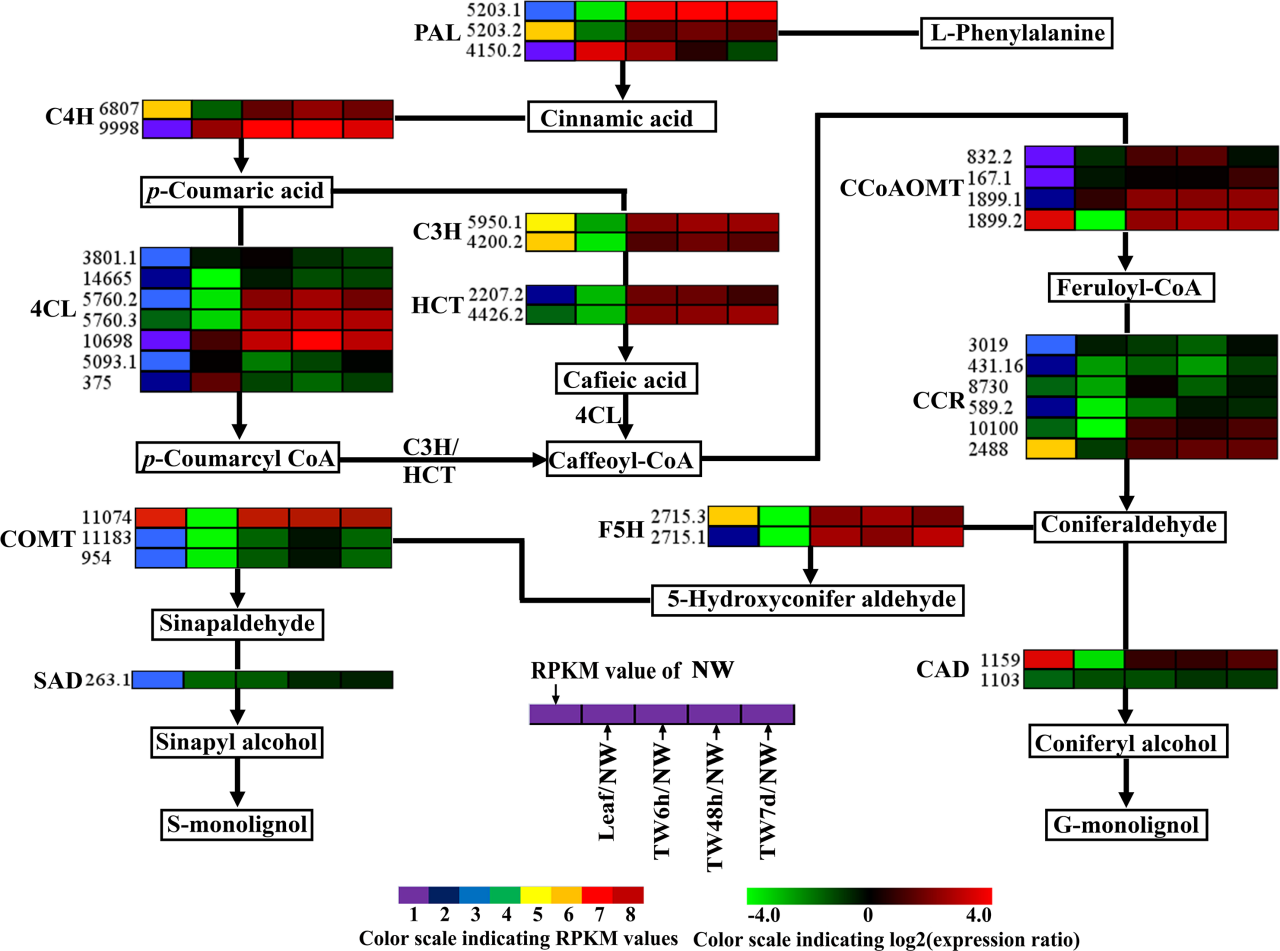
Expression patterns of DEGs related to the monolignol biosynthesis pathway. Enzyme names, Unigene ids and expression patterns are indicated on each step. The expression patterns of each Unigene are shown by five grids, with the left representing the RPKM value of the NW sample and the second to fifth representing the relative log2 (expression ratio) for the leaf, TW6h, TW48h and TW7d samples, from left to right. The grids with eight different colors from purple to red show the absolute expression magnitude of the NW sample, with RPKM values of 0–10, 10–20, 20–40, 40–80, 80–160, 160–320, 320–640 and over 640.

TW is characterized by a relatively higher cellulose content and a lower degree of lignification (Foston et al., 2011; Pilate et al., 2004; Wada et al., 1995); therefore expression analysis of genes involved in cellulose and lignin biosynthesis could provide a basis for elucidating the molecular mechanism of TW formation. In total, 125 Unigenes encoding 21 enzymes related to cellulose and lignin biosynthesis were expressed at the early stages of TW formation, and 77 of them were differentially expressed, including 42 up-regulated genes, 29 down-regulated genes and six genes without clear expression patterns (Tables 1 and 2 and Table S5). Among these DEGs, 43 (55.8%) Unigenes were preferentially expressed in NW (Tables 1 and 2). In the biosynthetic pathway of cellulose, 15 Unigenes encoding the related enzymes presented significantly up-regulated expression (Fig. 5, Table 1 and Table S5), which could be attributed to the fact that TW produces the cellulose-rich G-layer. Specifically, five Unigenes (4901.4, 4901.5, 4901.7, 824.3 and 824.4) encoding CesA and SUS exhibited dramatic up-regulation (—log_2_(TW/NW)— ≥4) and were strongly expressed in the developing xylem. In the monolignol biosynthesis pathway, 66 related Unigenes were identified, and six Unigenes annotated as CCR, CAD, and SAD were apparently down-regulated (Fig. 6, Table 2 and Table S5). In contrast, 27 genes were significantly up-regulated during the early stages of TW formation (Fig. 6, Table 2 and Table S5). For example, the transcripts of eight DEGs annotated as *PAL*, *C4H*, *C3H* and *HCT* were increased significantly, seven of which were preferentially expressed in the NW compared with Leaf (Fig. 6).

**Table 1:** Candidate genes involved in cellulose biosynthesis related metabolism.

**Gene**	**Enzyme**	**KO id (EC-No.)**	**No. All**^a^	**No. Change**^b^	**No. Up**^c^	**No. Down**^d^	**No.Xylem**^e^
*CesA*	Cellulose synthase	K10999 (EC:2.4.1.12)	21	13	7	5	5
*SUS*	Sucrose synthase	K00695 (EC:2.4.1.13)	7	2	2	0	2
*GTF*	Glucosyltransferase	K05841 (EC:2.4.1.173)	4	0	0	0	2
*UGP*	UDP-Glucose Pyrophosphorylase	K00963 (EC:2.7.7.9)	3	3	2	1	1
*PGM*	Phosphoglucomutase	K01840 (EC:5.4.2.8)	5	3	1	2	0
*HK*	Hexokinase	K00844 (EC:2.7.1.1)	5	2	1	1	0
*FRK*	Fructokinase	K00847 (EC:2.7.1.4)	9	6	1	5	3
*KOR*	endo-1,4-beta-glucanase	K01179 (EC:3.2.1.4)	5	5	1	1	2

**Notes.**

a No. All indicates the total number of Unigenes analyzed.

b No. Change indicates the number of Unigenes with expression significantly changed during tension wood formation.

c No. Up indicates the number of Unigenes with expression significantly up-regulated during tension wood formation.

d No. Down indicates the number of Unigenes with expression significantly down-regulated during tension wood formation.

e No. Xylem indicates the number of DEGs preferentially expressed in the xylem (${\log }_{2} \frac{\text{Leaf}}{\mathrm{NW}} \lt -1$).

**Table 2:** Candidate genes involved in lignin biosynthesis related metabolism.

**Gene**	**Enzyme**	**KO id (EC-No.)**	**No. All**^a^	**No. Change**^b^	**No. Up**^c^	**No. Down**^d^	**No.** **Xylem**^e^
*PAL*	Phenylalanine ammonia lyase	K10775 (EC:4.3.1.24)	3	3	2	0	2
*C4H*	Cinnamate 4-hydroxylase	K00487 (EC:1.14.13.11)	2	2	2	0	1
*4CL*	4-coumarate CoA ligase	K01904 (EC:6.2.1.12)	11	7	3	4	3
*C3H*	p-coumarate 3-hydroxylase	K05280 (EC:1.14.13.21)	2	2	2	0	2
*HCT*	p-hydroxycinnamoyl CoA shikimate /quinate p-hydroxycinnamoyl transferase	K13065 (EC:2.3.1.133)	2	2	2	0	2
*CCoAOMT*	Caffeoyl CoA O-methyltransferase	K00588 (EC:2.1.1.104)	5	4	3	0	1
*F5H*	Ferulate 5-hydroxylase	K09755 (EC:1.14.-.-)	2	2	2	0	2
*COMT*	Caffeic acid O-mothyltransferase	K05279 (EC:2.1.1.76)	7	3	1	2	3
*CCR*	Cinnamoyl CoA reductase	K09753 (EC:1.2.1.44)	14	6	2	4	4
*CAD*	Cinnamyl alcohol dehydrogenase	K00083 (EC:1.1.1.195)	5	2	1	1	2
*SAD*	Sinapyl alcohol dehydrogenase	K00083 (EC:1.1.1.195)	1	1	0	1	1
*SAMS*	S-adenosylmethionine synthetase	K00789 (EC:2.5.1.6)	5	5	5	0	3
*POD*	Peroxidase	K00430 (EC:1.11.1.7)	7	4	2	2	2

**Notes.**

a No. All indicates the total number of Unigenes analyzed.

b No. Change indicates the number of Unigenes with expression significantly changed during tension wood formation.

c No. Up indicates the number of Unigenes with expression significantly up-regulated during tension wood formation.

d No. Down indicates the number of Unigenes with expression significantly down-regulated during tension wood formation.

e No. Xylem indicates the number of DEGs expressed preferentially in the xylem (${\log }_{2} \frac{\text{Leaf}}{\mathrm{NW}} \lt -1$).

**Figure 7:**
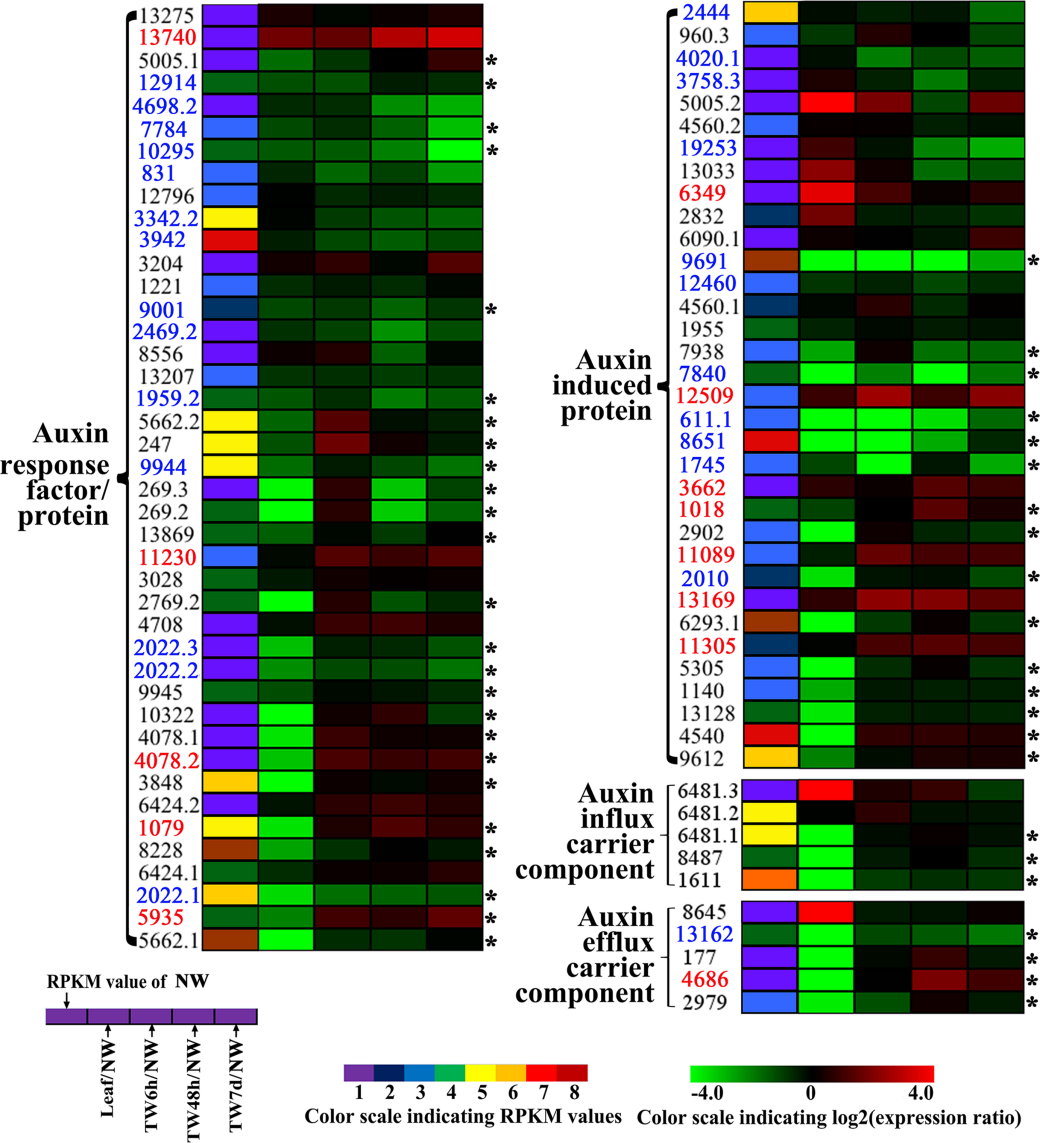
Clustering and identities of genes involved in polar transport and the related auxin response based on gene expression. Unigenes preferentially expressed in NW when compared to leaf are marked by the asterisk. The names of those up- and down-regulated Unigenes are indicated in red and blue, respectively.

Indole acetic acid (auxin) is a key regulator of wood development (Nilsson et al., 2008). The expression patterns of auxin-regulated genes in this study are shown in Fig. 7. In total, 42 Unigenes annotated as auxin-responsive factors (ARFs) were expressed during the bending treatment (Table S6), 25 of these ARFs were preferentially expressed in NW when compared to Leaf (Fig. 7). Compared with NW, 26 (61.9%) DEGs were found, including 14 down-regulated Unigenes (Fig. 7). It is worth noting that the transcript levels of 13 Unigenes and 18 Unigenes were changed significantly after 6 h and 48 h of bending treatment, respectively. Among the 34 Unigenes annotated as auxin-induced proteins (Fig. 7 and Table S6), 22 Unigenes presented the significant expression changes, including seven up-regulated and 11 down-regulated Unigenes (Fig. 7 and Table S6). Furthermore, when 6 h and 48 h of bending were applied, 12 and 16 DEGs in TW were detected, respectively. In addition, 10 Unigenes encoding auxin-transport carrier proteins were expressed over the period of TW formation, and only three Unigenes annotated as efflux carrier proteins presented obvious expression changes including one up-regulated gene (Fig. 7, Table S6).

### Identification of genes co-expressed with cellulose synthesis genes

To derive useful biological information, 12 up-regulated DEGs coding for CesA, SUS, KOR, UGP and FRK were selected as “guide genes” to identify co-expressed genes using RPKM data from five libraries. According to the MR method, a co-expression network was built based on the 20 most strongly correlated genes for each guide gene, and 215 Unigenes with similar expression trends were examined (Fig. S5). Of 198 Unigenes with annotations, 18 Unigenes coded for transcription factors (TFs), such as MYB TFs, Knotted1-likehomebox (KNOX) TFs and OVATE family proteins (OFPs) (Table S7). For instance, Unigene 3087 encoding a MYB TF was mainly expressed in the xylem and significantly increased during the early stages of TW formation (Table S7). The expression of Unigene 3087 was highly similar to that of *BlCesA1* (Unigene 4901.5). Furthermore, Unigenes 13166 and 19576 were both annotated as OFPs, and their expression patterns were similar to those of Unigene 4487 (*BlKOR1*) and Unigene 6504.2 coding for UGP, respectively (Table S7).

### Validation of the gene expression profiles by qRT-PCR

**Figure 8:**
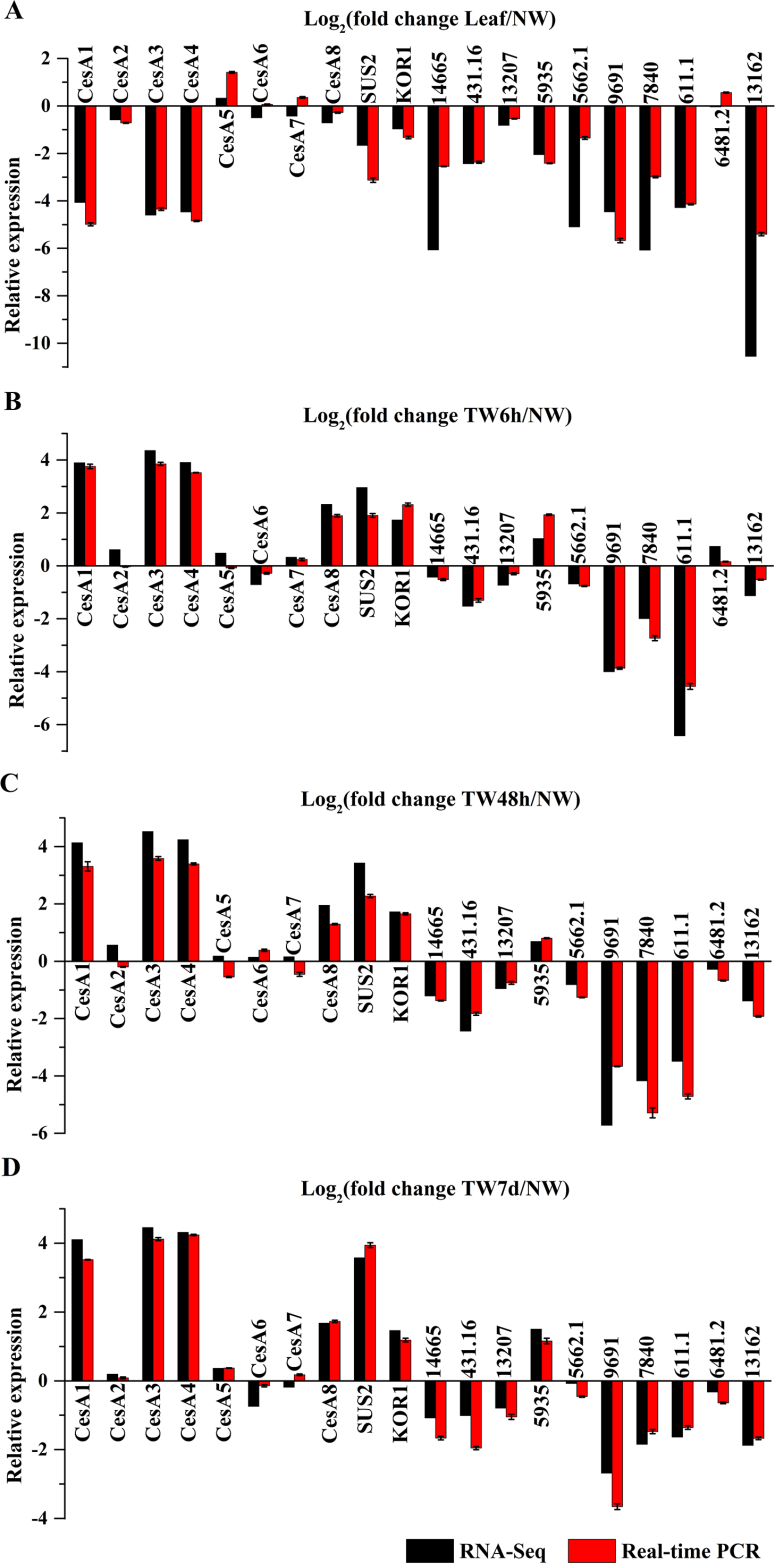
Confirmation of the expression profiles of 20 genes in *B. luminifera* by quantitative reverse transcription polymerase chain reaction (qRT-PCR). (A) Expression comparisons of selected genes detected by RNA-Seq and qRT-PCR between Leaf and NW. (B) Expression comparisons of selected genes detected by RNA-Seq and qRTPCR between TW6 h and NW. (C) Expression comparisons of selected genes detected by RNA-Seq and qRT-PCR between TW48h and NW. (D) Expression comparisons of selected genes detected by RNA-Seq and qRT-PCR between TW7d and NW. The y-axes show log2 (fold differences) determined by RNA-Seq and qRT-PCR. Error bars indicate standard deviations.

To validate the gene expression profiles, 20 gene*s* were selected for the transcript analysis by qRT-PCR, and the results were compared to the RNA-Seq data (Fig. 8). As a result, the expression of 15 genes (*BlCesA1*, *BlCesA3*, *BlCesA4*, *BlCesA8*, *BlSUS2*, *BlKOR1*, Unigene14665, Unigene431.16, Unigene13207, Unigene5935, Unigene5662.1, Unigene9691, Unigene7840, Unigene611.1 and Unigene13162) generally agreed with the results of RNA-Seq results (Fig. 8). While, the expression levels of *BlCesA2*, *BlCesA5*, *BlCesA6*, *BlCesA7* and Unigene6481.2 exhibited inconsistency in some samples, and the fold differences were all less than one (Fig. 8). These results showed similar expression patterns for most genes between RNA-Seq and qRT-PCR, and indicated the reliability of the RNA-Seq data.

## Discussion

Due to its high throughput and cost-effectiveness, next-generation sequencing technology, such as Illumina sequencing technology, has been widely applied to determine the transcriptomes of nonmodel plants. In recent years, many transcriptome data on nonmodel plants, including *Cunninghamia lanceolata* (Huang et al., 2012; Qiu et al., 2013), *Camellia sinensis* (Shi et al., 2011), Chinese bayberry (Feng et al., 2012), *Calotropis gigantea* (Muriira et al., 2015) and *Periploca sepium* (Zhang et al., 2017), have been generated using Illumina sequencing technology. These transcriptome data provide valuable resources for further studies in these plants. In this study, a transcriptome dataset of *B. luminifera* containing 45,700 assembled Unigenes was obtained using Illumina paired-end sequencing technology. Additionally, more than 75% of the Unigenes were found to have homologs in the four public databases, which provides important information about the functions of these genes in *B. luminifera*. In addition, 27,531 and 12,224 Unigenes were assigned to GO terms and COG classifications, respectively, and 19,474 Unigenes were mapped to 128 KEGG pathways. These results indicate that a wide diversity of transcripts was present in our reference transcriptome dataset, which reflects the high coverage of the transcriptome data and would enhance the probability of the identification of those genes involved in specific biological processes.

Reaction wood, a special wood induced by gravistimulation, has been used as a research model to identify the key genes involved in wood formation. For instance, 196 genes differentially regulated in the xylem of *Eucalyptus* were identified during tension wood formation (Paux et al., 2005). In *Populus*, those genes responsible for carbon flow between various cell wall components were identified through global transcription analysis and are believed to be important for G-layer formation in tension wood (Andersson-Gunneras et al., 2006). Additionally, 331 up-regulated and 165 down-regulated genes were identified in maritime pine (*Pinus pinaster*) during the differentiation of compression wood, which provides an important basis for further analysis of genes involved in the biosynthesis of different cell wall polymers associated with pine wood structure and composition (Villalobos et al., 2012). Nevertheless, little attention has been paid to the gene expression profiles of the secondary xylem during TW formation in *B. luminifera*. In this study, based on the transcriptome dataset, 13,273 DEGs were identified during the early stages of TW formation, which implied that there were considerable changes in gene expression in the secondary xylem during this process. Furthermore, the GO functional classification showed that more up-regulated DEGs were assigned to the ‘metabolic process’ and ‘catalytic activity’ groups, implying that the metabolic processes enhanced by these DEGs might be important for TW formation in *B. luminifera*. These results provide valuable clues for elucidating the molecular mechanism underlying wood formation.

One of the main characteristics of TW is the occurrence of the G-layer and the reduced lignin content, as shown in *B. luminifera* (Fig. 2) and other hardwood species, such as poplar and eucalyptus. Thus, Unigenes responsible for cellulose and lignin biosynthesis should be critical for TW formation in angiosperms. As expected, over 60% of such Unigenes were differentially expressed at the early stages of TW formation (Tables 1 and 2), and more than half of the DEGs were preferentially expressed in NW when compared to Leaf (Figs. 5 and 6). This was similar to previous studies in *Pinus pinaster* and *Eucalyptus globulus* (Paux et al., 2005; Plomion et al., 2000), suggesting that cellulose and lignin biosynthesis in TW are regulated by some specific genes.

In the cellulose biosynthesis pathway, the Unigenes encoding cellulose synthase (CesA) were first analyzed in this study. Three *BlCesA* genes, i.e., *BlCesA1* (Unigene 4901.5), *BlCesA3* (Unigene 4901.4) and *BlCesA4* (Unigene 4901.7), were found to be significantly up-regulated at the early stages of TW formation in *B. luminifera* (Fig. 5). This significant up-regulation of *PttCesA3-2* and *EgCesA* was also observed in the induced TW of *Populus tremula* × *Populus tremuloides* and *Eucalyptus globulus* (Andersson-Gunneras et al., 2006; Djerbi et al., 2005). Interestingly, *BlCesA1* and *PttCesA3-2* were both homologous genes of *AtCesA8*, which implied that these *CesA* genes may play important roles in TW formation in hardwood trees (Fig. S6A) (Huang et al., 2014). The membrane-bound endo-*β*-1,4-glucanase (KOR) has been proposed to play an important role in assembling cellulose chains into microfibrils (Molhoj, Pagant & Hofte, 2002; Takahashi et al., 2009). The *PtdKOR* of **P. tremuloides**, a homologous gene of *AtKOR1*, was significantly up-regulated in the aspen stem in response to tension stress and showed similar expression pattern to *PtdCesA1*, *PtdCesA2*, and *PtdCesA3* (Bhandari et al., 2006). In this study, *BlKOR1* (Unigene 4487) was also a homologous gene of *AtKOR1* (Fig. S6B ), and its expression was significantly up-regulated during the early stages of TW formation (Fig. 5). Interestingly, *BlKOR1* was also co-expressed with *BlCesA1*, *BlCesA3*, and *BlCesA4*, and these genes were all strongly expressed in the secondary xylem (Fig. 5). These co-expressed genes might be responsible for the synthesis of microfibrils in TW. Sucrose transport is the central system for the allocation of carbon (C) resources in vascular plants, which is important for wood formation. SUS is a key enzyme controlling the sucrose transport system (Zheng et al., 2011), and it catalyzes the hydrolysis of sucrose to generate UDP-glucose (i.e., the substrate of the CSC). *BlSUS2* (Unigene 824.3 and 824.4) was significantly up-regulated during TW formation. Similarly, its homologs in *P. tremula* × **P. tremuloides** (Fig. S6C ), *PttSUS1* and *PttSUS2* showed much higher transcript levels in TW compared to NW (Andersson-Gunneras et al., 2006). This means that the carbon allocation mediated by SUS is probably a critical step in TW formation. Another consequence of sucrose hydrolysis is the accumulation of fructose in the cytoplasm. A high level of fructose will activate a sequential fructose metabolic process, of which the phosphorylation of fructose catalyzed by FRK is the first step. In the TW of *B. luminifera*, a significant increase in fructose concentrations was detected during TW formation (Fig. 3B), and the expression of one *FRK* (Unigene 572) was significantly up-regulated with increasing fructose (Fig. 5), suggesting that the accumulation of fructose might be another important event during TW formation. These results implied that the higher level of *BlSUS2* may facilitate more C transport to the xylem, which would lead to the accumulation of more cellulose in TW. In addition, the expression levels of several Unigenes encoding HK, PGM and UGP in TW significantly increased (Fig. 5), which further supports our hypothesis of the increased C flux in TW for the biosynthesis of cellulose.

For monolignol biosynthesis, 66 related Unigenes were found to be expressed at the early stages of TW formation in *B. luminifera* (Table 2). In the monolignol-specific pathway, most Unigenes annotated as CCR, CAD, and SAD displayed an obviously down-regulated expression (Fig. 6), which is consistent with the relative decrease in lignin content in TW. In addition, three DEGs encoding F5H and COMT, which directly associate with the synthesis of S-monolignol, showed significantly up-regulated expression in TW (Fig. 6). Meanwhile, the expression levels of *BlSAMS2* (Unigene 2887.1) and *BlSAMS3* (Unigene 2887.4) were significantly up-regulated compared with NW in our study (Table 2 and Table S5), which may indirectly provide more methyl groups for the metabolic step catalyzed by COMT (Meng & Campbell, 1998). This finding implied that bending treatment might promote the accumulation of S-monolignol in the TW of *B. luminifera*. Joseleau et al. (2004) reported that S-monolignol was abundant in the newly formed G-layer of TW in *Populus deltoides*. For this reason, it can be speculated that although the total content of lignin in *B. luminifera* TW was apparently lower than that of NW, the S:G ratio of lignin might increase comparatively. On the other hand, it is worth noting that many Unigenes encoding for PAL, C4H, C3H, and HCT, which were not directly responsible for the synthesis of monolignols, exhibited significant up-regulation at the early stages of TW formation (Fig. 6). This does not agree with a recent study in *Eucalyptus* that stated that changes in cell wall traits induced by TW formation resulted from the down-regulation of lignin biosynthesis genes (Mizrachi et al., 2015). However, the metabolites of this pathway include not only lignin but also tannin and flavonoid. Therefore, these Unigenes might be responsible for the formation of tannin and flavonoid but not monolignols, and their expression could be triggered by bending treatment. Nevertheless, further research is necessary to elucidate the roles of these enzymes in the biosynthesis of lignin.

Co-expression analysis, which is based on the assumption that genes with similar expression patterns are more likely to be functionally associated, has been proven to be a powerful tool for identifying regulatory factors in transcriptional networks (Persson et al., 2005). In this study, 18 TFs were identified to be co-expressed with biosynthetic genes of the cellulose biosynthesis pathway during TW formation (Fig. S5 and Table S7). Among these TFs, MYB transcription factors have been indicated as important regulators for wood formation (Xu et al., 2017; Li et al., 2015; Petzold et al., 2018; Soler et al., 2017; Soler et al., 2016). For example, *PtiMYB75* could functionally activate the expression of the biosynthetic genes involved in cellulose, lignin and hemicellulose biosynthesis (Zhong et al., 2011). The functional study revealed that *EgMYB88* could control the biosynthesis of some phenylpropanoid-derived secondary metabolites including lignin in cambium and in the first layers of differentiating xylem (Soler et al., 2016). In our study, Unigene 3087, a homolog of *PtiMYB75* (Fig. S7A), was found to be co-expressed with *BlCesA1* (Unigene 4901.5) (Fig. S5) and preferentially expressed in the secondary xylem (Table S7). These results reveal the important roles of MYB transcription factors in the regulation of cellulose and lignin biosynthesis during wood formation. Another important TF identified by co-expression analysis is OFP, which is one family of the plant-specific family of regulatory proteins that have been demonstrated to play important roles in the regulation of plant growth and development (Cong, Liu & Tanksley, 2002; Hackbusch et al., 2005; Nesbitt & Tanksley, 2002; Wang, Chang & Ellis, 2016). Unigenes 13166 and 19576, two homologs of AtOFPs (Fig. S7B ), were both preferentially expressed in the secondary xylem and co-expressed with the cellulose biosynthesis-related genes Unigene 4487 (*BlKOR1*) and Unigene 6504.2 (UGP), respectively (Figs. S5 and S7). In *Arabidopsis thaliana*, AtOFP1 and AtOFP4 have been shown to physically interact with KNAT7 and BLH6, and were suggested to be important components of a putative multiprotein transcription regulatory complex containing BLH6 and KNAT7, which play critical roles in secondary cell wall formation (Liu & Douglas, 2015; Li et al., 2011). However, no other studies about the regulatory role of OFP in growth and development, especially in wood formation, have been reported in woody plants. Therefore, further studies are required on the regulatory functions of these two *OFP* genes in TW formation of *B. luminifera*.

According to previous reports, reaction wood is induced by a redistribution of IAA around the stem (Hellgren, Olofsson & Sundberg, 2004; Mellerowicz et al., 2001; Robnett & Morey, 1973). In hybrid aspen (*P. tremula* × **P. tremuloides**), the expression levels of specific *PttIAA* genes were differentially altered after 24 h of bending, but no measurable change in auxin content of the xylem was detected (Moyle et al., 2002). This suggests that under gravistimulation, either auxin distribution or auxin sensitivity in the xylem cells could be altered and lead to the initiation of TW formation (Moyle et al., 2002). In another report, Hellgren, Olofsson & Sundberg (2004) also discovered that there were no obvious alterations in endogenous IAA levels across the cambial region tissues of *P. tremula* and *Pinus sylvestris* forming reaction wood. In our study, more than half of the annotated Unigenes involved in the auxin signaling pathway in *B. luminifera* exhibited differential expression in TW, and most of them showed a tendency to be down-regulated (Fig. 7). However, it was only after 7 d of bending that a significant reduction in IAA concentration in TW was observed in our study (Fig. 3C). This, to an extent, demonstrated that TW initiation of *B. luminifera* could not be attributed mainly to the decrease in the IAA content. The expression levels of Unigenes encoding auxin influx carrier components did not show significant changes, but one efflux carrier gene (Unigene 4686) encoding PIN1-like protein was significantly up-regulated (Fig. 7, Table S6), indicating that auxin efflux might occur in the TW xylem. Recently, a model for gravitropism in the woody stem was proposed suggesting that the peripheral location of PIN3-expressing cells relative to the cambium results in auxin transport towards the cambium in the upper region of the stem, triggering TW formation (Gerttula et al., 2015). In addition, exogenous IAA treatment in hybrid poplar also indicated that TW formation is closely associated with the acceleration of intercellular auxin polar transport (Min et al., 2017). Taken together, our results suggest that during the early stages of *B. luminifera* TW formation, there is a tendency of the IAA content in TW to decrease, or there is an auxin concentration gradient across the radially arranged secondary vascular tissues, initiating TW formation.

## Conclusions

In this study, a reference transcriptome dataset of *B. luminifera* was first generated containing 45,700 Unigenes, and 35,135 (76.9%) Unigenes were annotated by a BLAST similarity search against four public databases. The global expression pattern and differentially expressed genes during the early stage of TW formation were then identified in *B. luminifera* using RNA-Seq. In total, 13,273 DEGs were identified for TW formation, especially for those involved in the biosynthesis of cellulose and lignin in the secondary cell wall and auxin signaling. Furthermore, through co-expression analysis, 18 TFs were identified to be associated with the regulation of cellulose biosynthesis in TW formation. The high-resolution transcriptome profiles produced and the differentially expressed genes identified in this study could be used to improve our understanding of the molecular mechanisms in wood formation of tree species, particularly in *B. luminifera*, and could be explored for molecular breeding of wood quality in *B. luminifera* using the candidate gene approach.

##  Supplemental Information

10.7717/peerj.5427/supp-1 Table S1 Sequence alignment of Unigenes involved in cellulose and lignin biosynthesis

10.7717/peerj.5427/supp-2 Table S2 Primer sequences for selected genes in this experiment

10.7717/peerj.5427/supp-3 Table S3 GenBank accession numbers of the other gene suquences used in phylogenetic analysis

10.7717/peerj.5427/supp-4 Table S4 KEGG pathway annotation of *B. luminifera* Unigenes

10.7717/peerj.5427/supp-5 Table S5 Annotations of DEGs involved in cellulose and lignin biosynthesis

10.7717/peerj.5427/supp-6 Table S6 Annotations of Unigenes involved in auxin polar transport and related response

10.7717/peerj.5427/supp-7 Table S7 RPKM values and annotations of co-expressed Unigenes with CesA, SUS, KOR, UGP and FRK during TW formation

10.7717/peerj.5427/supp-8 Figure S1 Length and sequencing depth distribution of assembled Unigenes from *B. luminifera* (A) Length distribution; (B) Sequencing depth distribution.

10.7717/peerj.5427/supp-9 Figure S2 GO classification of Unigenes with BLASTX matches against the NR database The left y-axis indicates the percentage of a specific category of genes in that main category. The right y-axis indicates the number of genes in the same category.

10.7717/peerj.5427/supp-10 Figure S3 COG Function Classification of the *B. luminifera* transcriptome

10.7717/peerj.5427/supp-11 Figure S4 The phenylpropanoid biosynthesis pathway (map 00940) in *B. luminifera* transcriptome is annotated through KEGG database The red box represented the enzymes discovered in the transcriptome data.

10.7717/peerj.5427/supp-12 Figure S5 Co-expression network of Unigenes in the cellulose biosynthesis pathway The yellow and purple circles represent the guide and co-expressed Unigenes, respectively. Unigene ids were indicated inside the circles.

10.7717/peerj.5427/supp-13 Figure S6 Neighbor-joining phylogenetic trees of the plant CesA, KOR and SUS protein sequences (A) Phylogenetic tree of 87 plant CESA proteins. Clades containing CESAs mainly associated with primary cell wall synthesis were denoted by a green background and clades linked to secondary cell wall synthesis were shown by a yellow background. (B) Phylogenetic tree of 13 plant KOR or KOR-like proteins. (C) Phylogenetic tree of 16 plant SUS proteins. Species names were abbreviated as At, *Arabidopsis thaliana*; Bl, *Betula luminifera*; Bp, *Betula platyphylla*; Cl, *Cunninghamia lanceolata*; Egra, *Eucalyptus grandis*; Gh, *Gossypium hirsutum*; Hv, *Hordeum vulgare*; Os, *Oryza sativa*; Pr, *Pinus radiata*; Pt, *Pinus taeda*; Ptd, *Populus tremuloides*; Pti, *Populus trichocarpa*; Ptt, *Populus tremula* ×* P. tremuloide*; St, *Solanum tuberosum*; Ta, *Triticum aestivum*; Ze, *Zinnia elegans*; and Zm, *Zea mays*.

10.7717/peerj.5427/supp-14 Figure S7 Phylogenetic trees of the plant MYB and OFP protein sequences (A) Phylogenetic tree of 42 plant MYB proteins. (B) Phylogenetic tree of 24 plant OFP or OFP-like proteins. Species names were abbreviated as: At, *Arabidopsis thaliana*; Eg, *Eacalyptus grandis*; Mt, *Medicago truncatula*; Pt, *Pinus taeda*; Pti, *Populus trichocarpa*; Ptt, *Populus tremula* ×* P. Tremuloide*; Sl, *Solanum lycopersicum*; Zm, *Zea mays*.

## Additional Information and Declarations

### Data Availability

The following information was supplied regarding data availability: The sequences are available at the NCBI Short Read Archive (SRA) via accession numbers SRP023510 and SRP051136.
