# Supplementary figures and images for: RNA-Seq analysis of differential gene expression in Betula luminifera xylem during the early stages of tension wood formation
Source: PeerJ. 2018 Aug 21;6:e5427. doi: 10.7717/peerj.5427 (PMC6108316; doi:10.7717/peerj.5427)

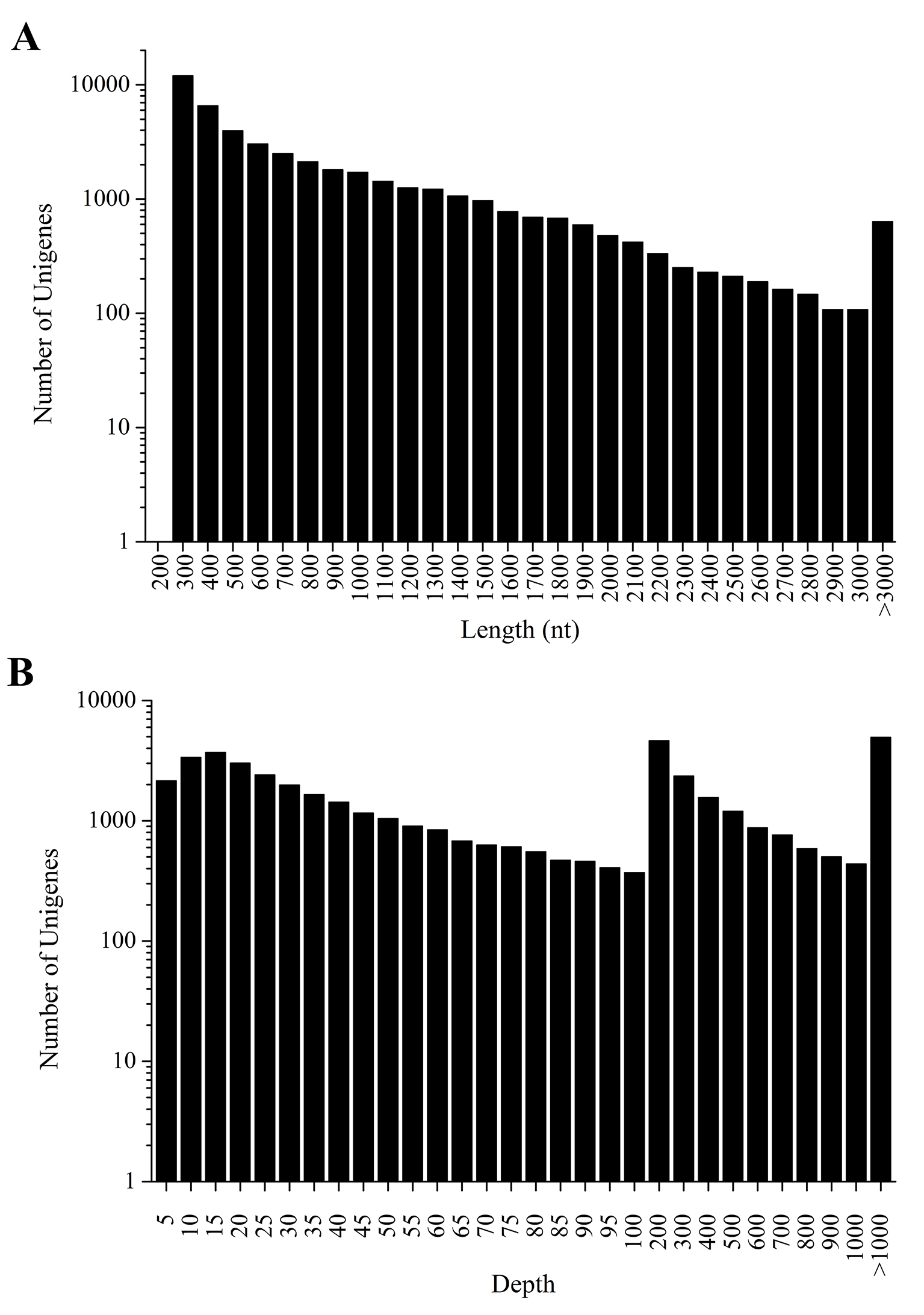

Supplement: Figure S1 — (A) Length distribution; (B) Sequencing depth distribution. [file peerj-06-5427-s008.png]

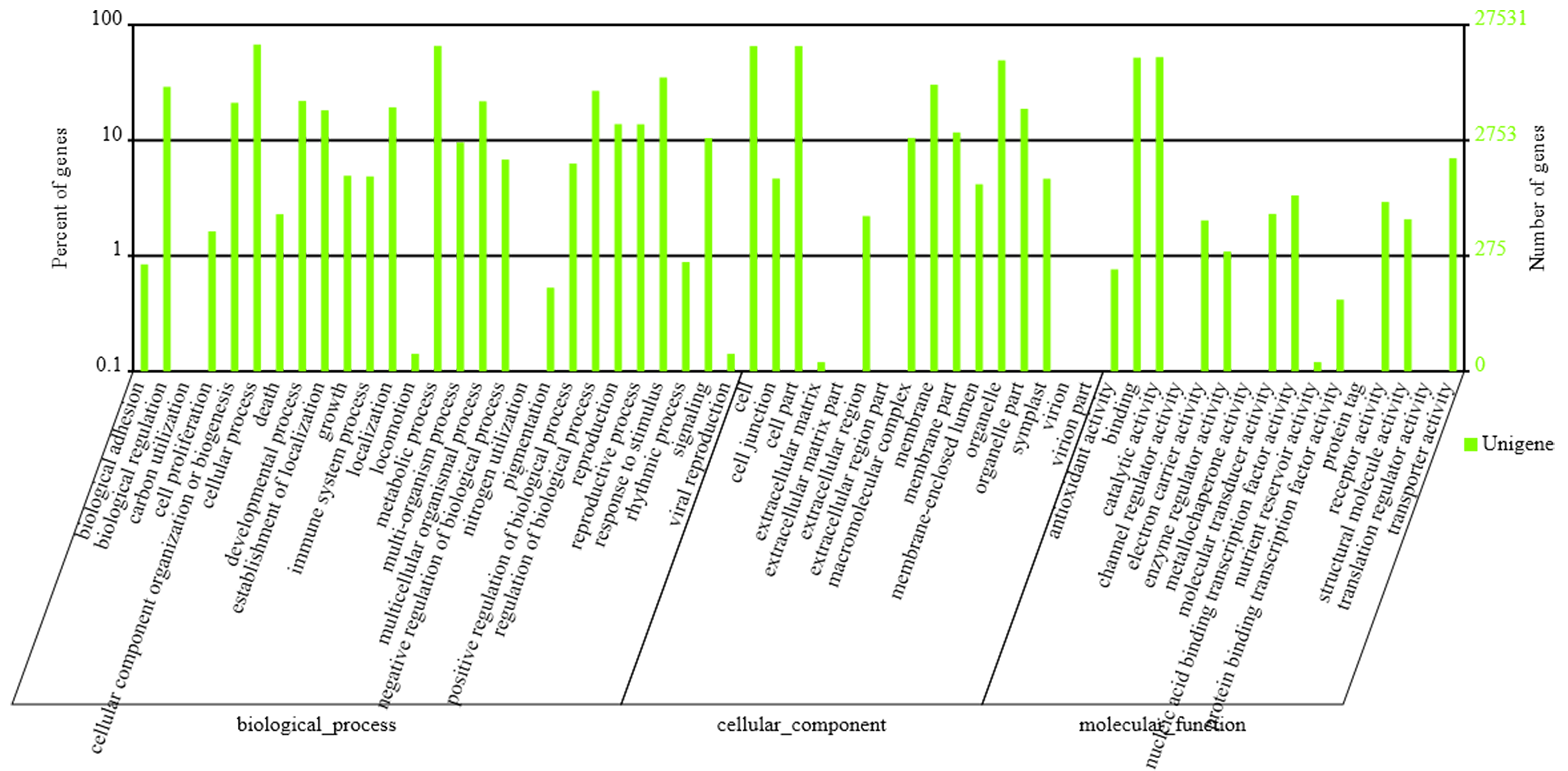

Supplement: Figure S2 — The left y-axis indicates the percentage of a specific category of genes in that main category. The right y-axis indicates the number of genes in the same category. [file peerj-06-5427-s009.png]

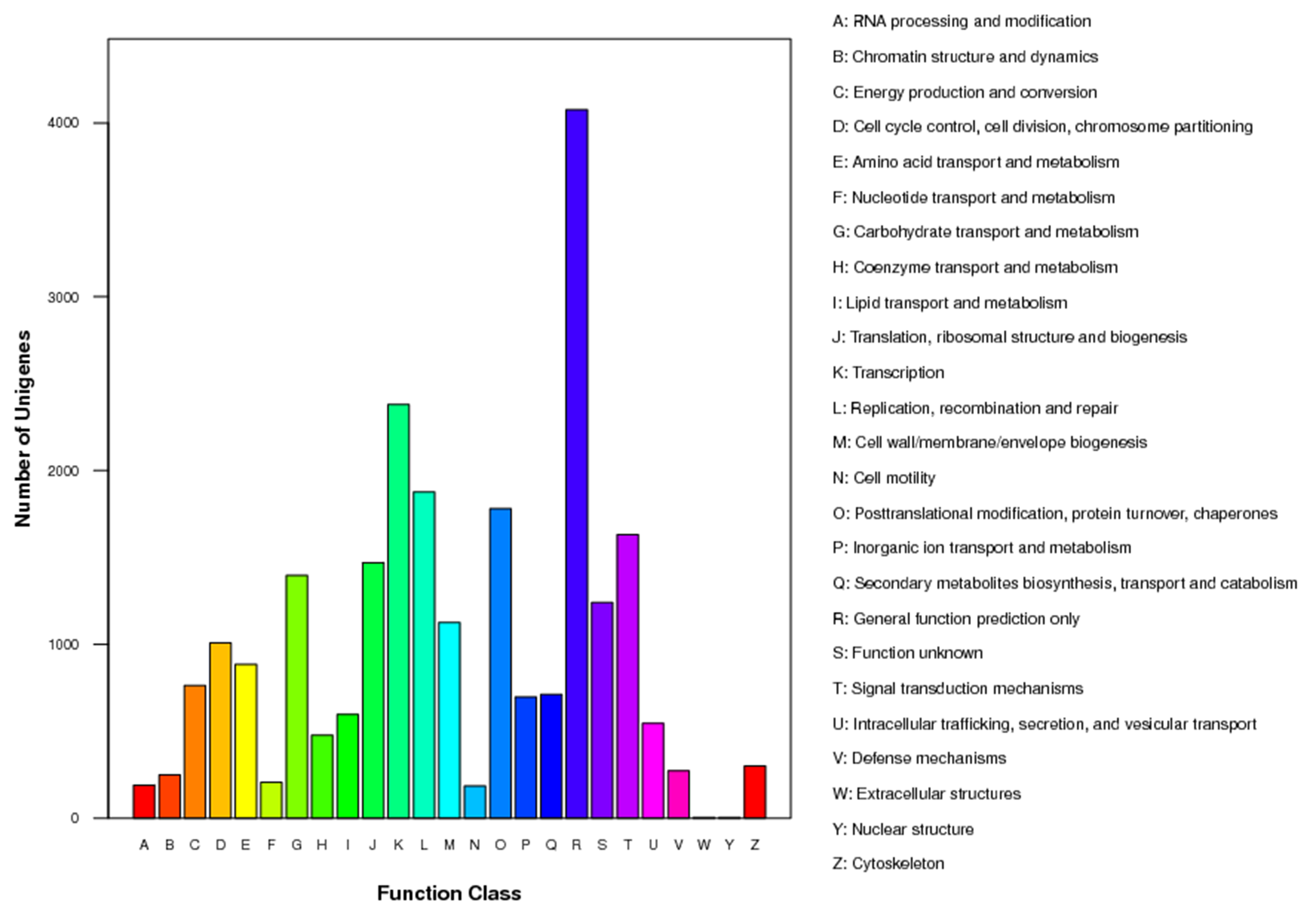

Supplement: Figure S3 [file peerj-06-5427-s010.png]

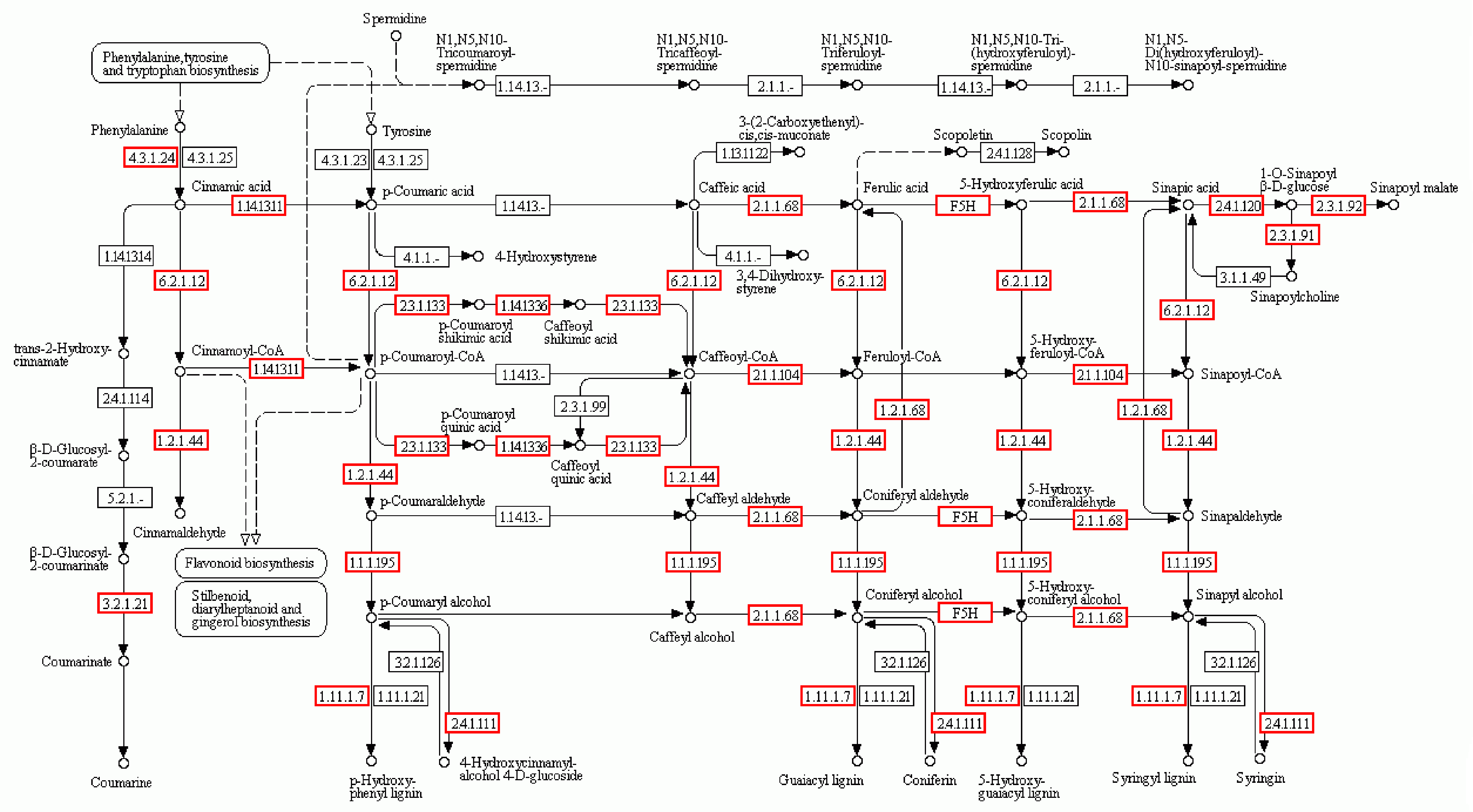

Supplement: Figure S4 — The red box represented the enzymes discovered in the transcriptome data. [file peerj-06-5427-s011.png]

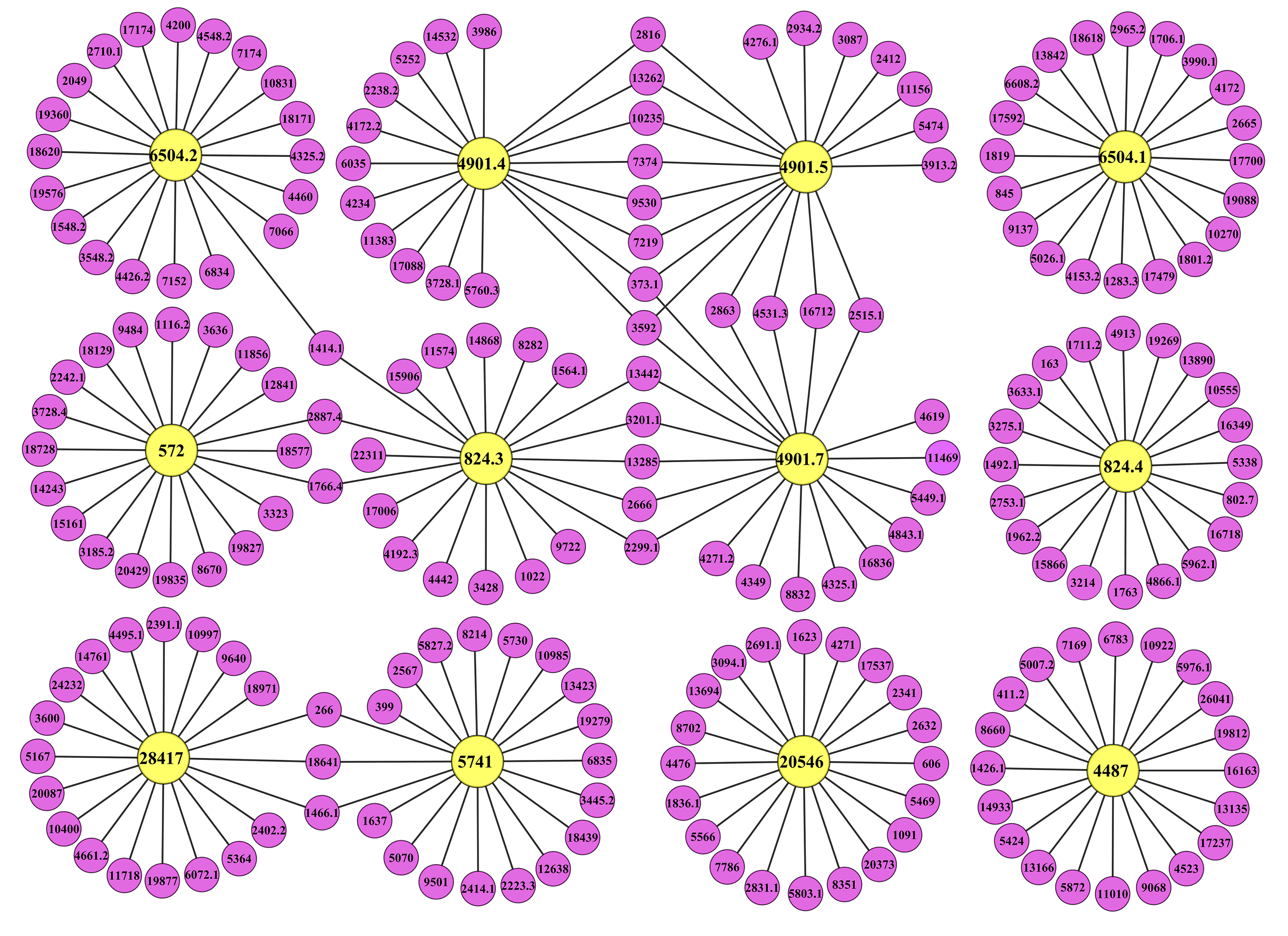

Supplement: Figure S5 — The yellow and purple circles represent the guide and co-expressed Unigenes, respectively. Unigene ids were indicated inside the circles. [file peerj-06-5427-s012.png]

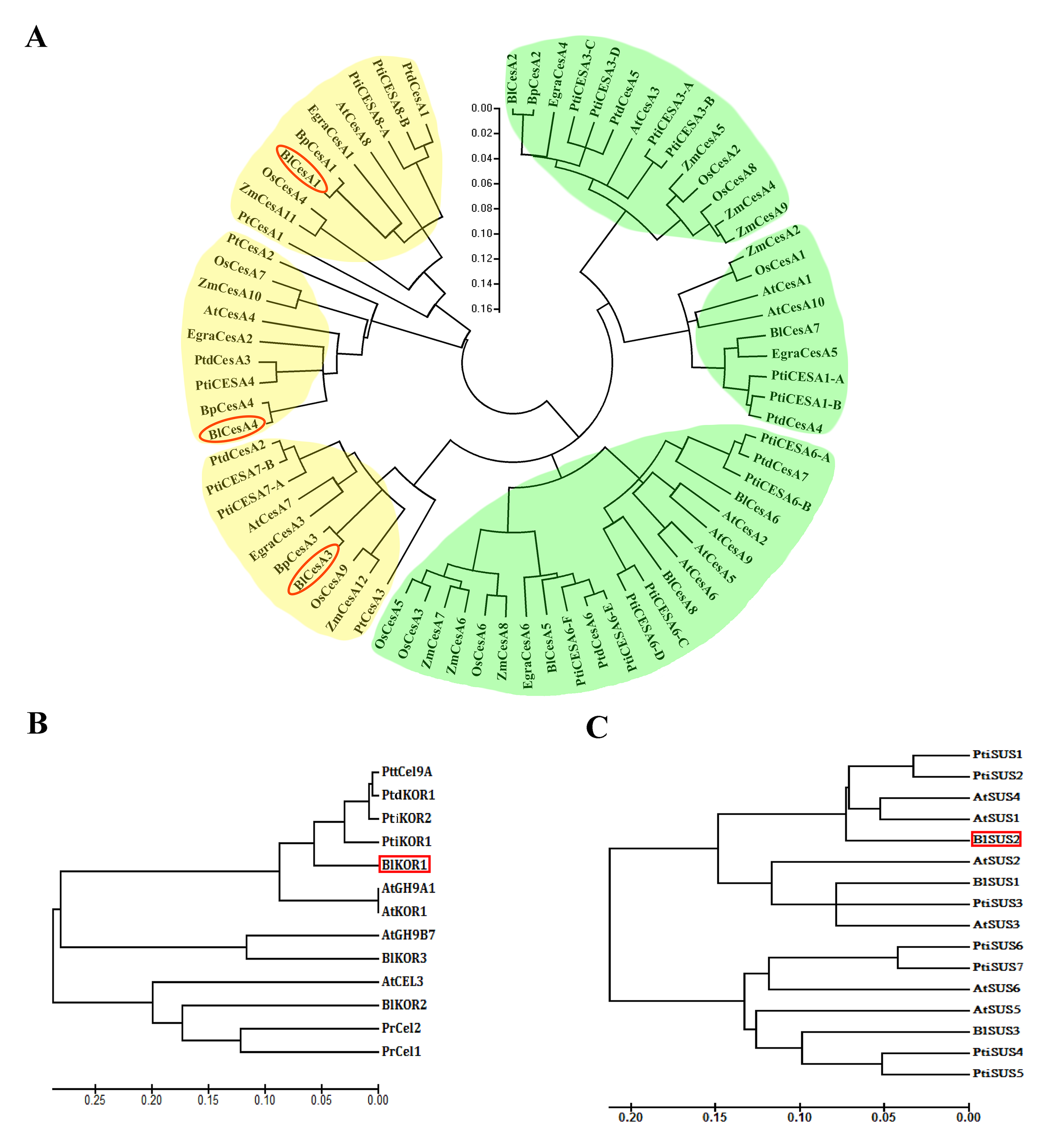

Supplement: Figure S6 — (A) Phylogenetic tree of 87 plant CESA proteins. Clades containing CESAs mainly associated with primary cell wall synthesis were denoted by a green background and clades linked to secondary cell wall synthesis were shown by a yellow background. (B) Phylogenetic tree of 13 plant KOR or KOR-like proteins. (C) Phylogenetic tree of 16 plant SUS proteins. Species names were abbreviated as At, Arabidopsis thaliana; Bl, Betula luminifera; Bp, Betula platyphylla; Cl, Cunninghamia lanceolata; Egra, Eucalyptus grandis; Gh, Gossypium hirsutum; Hv, Hordeum vulgare; Os, Oryza sativa; Pr, Pinus radiata; Pt, Pinus taeda; Ptd, Populus tremuloides; Pti, Populus trichocarpa; Ptt, Populus tremula × P. tremuloide; St, Solanum tuberosum; Ta, Triticum aestivum; Ze, Zinnia elegans; and Zm, Zea mays. [file peerj-06-5427-s013.png]

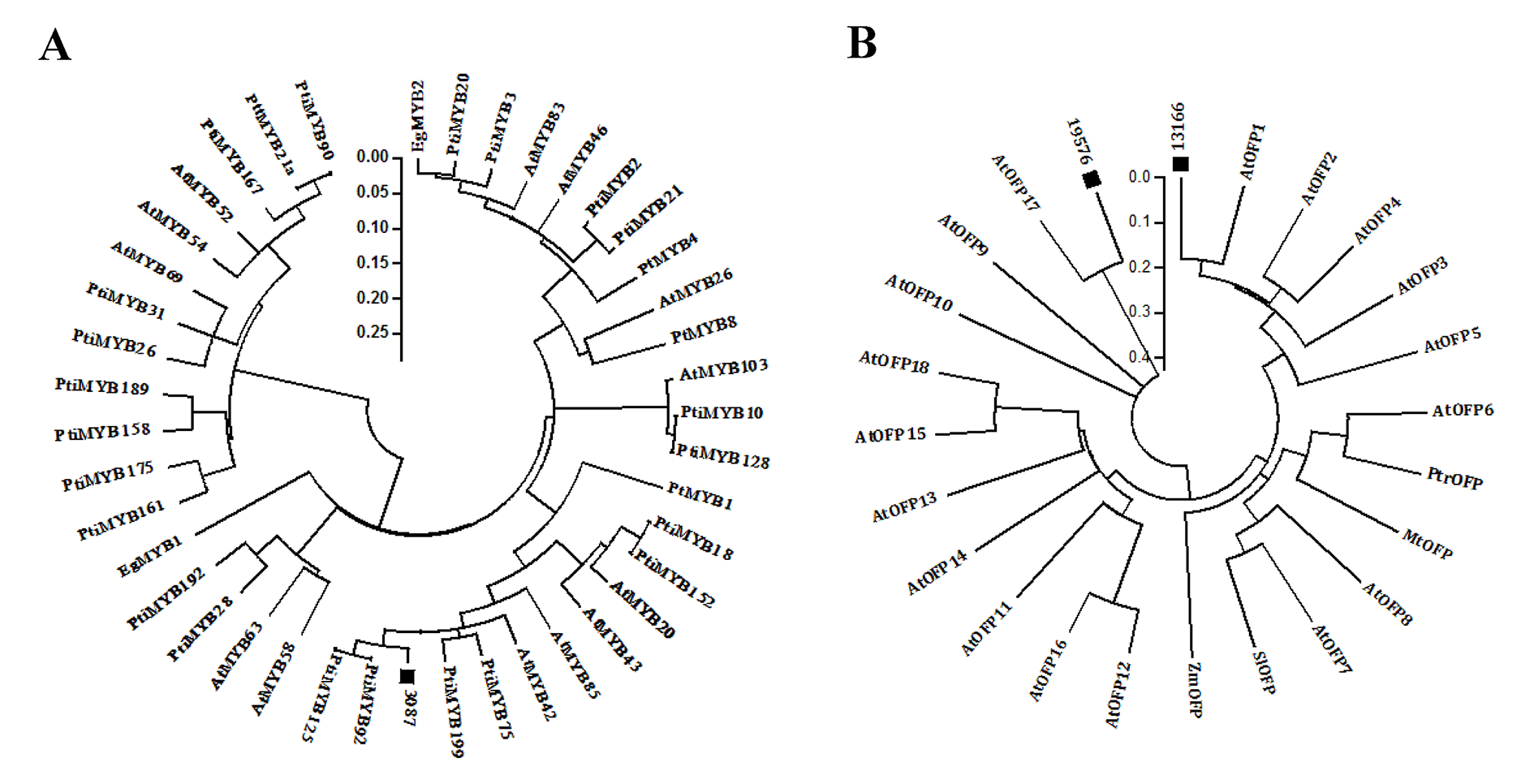

Supplement: Figure S7 — (A) Phylogenetic tree of 42 plant MYB proteins. (B) Phylogenetic tree of 24 plant OFP or OFP-like proteins. Species names were abbreviated as: At, Arabidopsis thaliana; Eg, Eacalyptus grandis; Mt, Medicago truncatula; Pt, Pinus taeda; Pti, Populus trichocarpa; Ptt, Populus tremula × P. Tremuloide; Sl, Solanum lycopersicum; Zm, Zea mays. [file peerj-06-5427-s014.png]
